# Nanobubble-Driven Precision Aeration: Deciphering Abiotic Hypoxia Responses via Multi-Omics for Next-Generation Smart Agriculture

**DOI:** 10.3390/antiox15101332

**Published:** 2026-10-15

**Authors:** Hafiz Abdul Kareem, Sana Saleem, Muhammad Bilal Hafeez, Naeem Ahmad, Da-Chuan Yin

**Affiliations:** 1 Key Laboratory for Space Bioscience and Biotechnology, School of Life Sciences, Northwestern Polytechnical University, Xi’an 710000, China; 2 College of Horticulture, Northwest A&F University, Yangling 712100, China; sanasaleem@nwafu.edu.cn; 3 College of Pastoral Agriculture Science and Technology, Lanzhou University, Lanzhou 730000, China; bilalhafeez32@gmail.com (M.B.H.); naeem@nwafu.edu.cn (N.A.)

**Keywords:** nanobubble water, oxygen nanobubbles, root-zone hypoxia, abiotic stress, multi-omics, rhizosphere oxygenation

## Abstract

Soil salinity and drought can restrict oxygen availability in the root zone, adding hypoxic stress to the ionic, osmotic, and hydraulic constraints experienced by crops. Nanobubble (NB) water offers a chemical-free approach for delivering highly dispersed oxygen and sustaining dissolved oxygen during irrigation. This review connects the physicochemical behavior of NBs with plant responses to salinity- and drought-associated hypoxia. It examines restoration of aerobic respiration and ATP homeostasis, modulation of ethylene response factor group VII and hypoxia-responsive gene signaling, recovery of ion transport and aquaporin function, redox regulation, hormonal cross-talk, and rhizosphere microbiome responses. Evidence from rice, maize, wheat, cotton, and controlled-environment systems is evaluated alongside opportunities for nanoparticle co-delivery, nanofertilizers, seed priming, and sensor-guided irrigation. The review also outlines how transcriptomics, proteomics, metabolomics, epigenomics, ionomics, and phenomics can resolve NB-mediated mechanisms across the plant–soil continuum. Current evidence supports NB irrigation as a promising precision-aeration platform, but field-scale validation, standardized NB characterization, dose–response studies, energy and cost assessments, and long-term environmental safety analyses remain necessary before broad agricultural deployment.

## 1. Introduction

### 1.1. The Escalating Abiotic Stress Crisis and Its Agricultural Consequences

Salinity and drought are two of the most serious abiotic challenges currently facing modern agriculture, and their combination, aggravated by climate warming, will prove a fundamental threat to global food security. Currently, salinity affects about 1.4 billion hectares (more than 10% of the terrestrial land surface), and an additional billion hectares is vulnerable due to climate change and unsustainable land management [1]. Trajectories are not favorable: by 2050, as much as 50% of the global arable land could be salt-affected, and this is already a reality: the portion of severely salinized land has been increasing by about 17% in the major agricultural basins, which account for 60% of global food production, between 2015 and 2023 [1,2]. The economic cost is also high, and yield losses caused by salinity are estimated at US$1.27–2.73 billion per year [3]. Drought stress alone significantly reduces grain yield and quality of cereal crops worldwide, affecting their nutritional properties and quality traits by modifying morphological, physiological, and biochemical traits in plants [4]. The two stresses are superficially different, but share common physiological lesions [5]. Salinity makes the soil potential (osmotic) negative, thereby causing excessive accumulation of Na^+^ and Cl^−^ in the soil, which causes ionic toxicity and production of excess reactive oxygen species (ROS), leading to disruption of cellular redox homeostasis [6]. Drought causes stomatal closure and a reduction in carbon assimilation, and the resulting decrease in leaf water potential slows root growth and seed production and forces metabolic reprogramming for osmotic adjustment [7]. More importantly, climate projections suggest that these stresses will become more likely to occur simultaneously and that they interact synergistically, meaning that when water and osmotic potential stresses occur simultaneously, they result in membrane integrity disruption and loss of control of the cellular machinery regulating water status [8,9,10].

### 1.2. Hypoxia: The Hidden Third Stressor

Beyond the extensively characterized ionic and osmotic dimensions of salinity and drought lies a mechanistically pivotal yet markedly understudied sequela: oxygen deprivation within the root zone, or rhizospheric hypoxia. This happens when the amount of molecular oxygen (O_2_) in the soil matrix and rhizosphere is below the level needed for the aerobic respiration of the roots. Although floods and waterlogging have conventionally been blamed for hypoxia, it is now known that this phenomenon is a downstream consequence of salinity and drought and occurs as a result of both separate and overlapping mechanisms. In addition, salinity causes physical compaction of soils, leading to reduced macroporosity and limiting O_2_ diffusion to the root surface [11,12], and salt effects on membrane permeability to O_2_ exacerbate the impact of increasing O_2_ depletion in the cell [13]. In a similar manner, drought causes compaction of the soil, thus decreasing gas exchange in the rhizosphere, and the simultaneous reduction in hydraulic conductivity of the roots hinders oxygen transport through the vascular continuum [14,15]. Regardless of its proximate cause, whether compaction, poor drainage, over-irrigation, or structural degradation, root-zone hypoxia is one of the most common and least explored limitations in agronomy. It slows down gas exchange at the rhizosphere, disrupts the metabolic activity of roots, changes the function of the microbial community, and reduces the enzyme activity that is considered to mediate nutrient turnover [16,17]. The physiological features that accompany the onset of salinity stress—fermentative catabolism, ATP depletion, cytoplasmic acidosis, ionic dysregulation, and ethylene production—create a positive feedback loop that strengthens and sustains the initial stress responses triggered by salinity and drought [18].

### 1.3. Nanobubble Water Technology: An Emerging Precision Oxygen Delivery Platform

**Figure 1:**
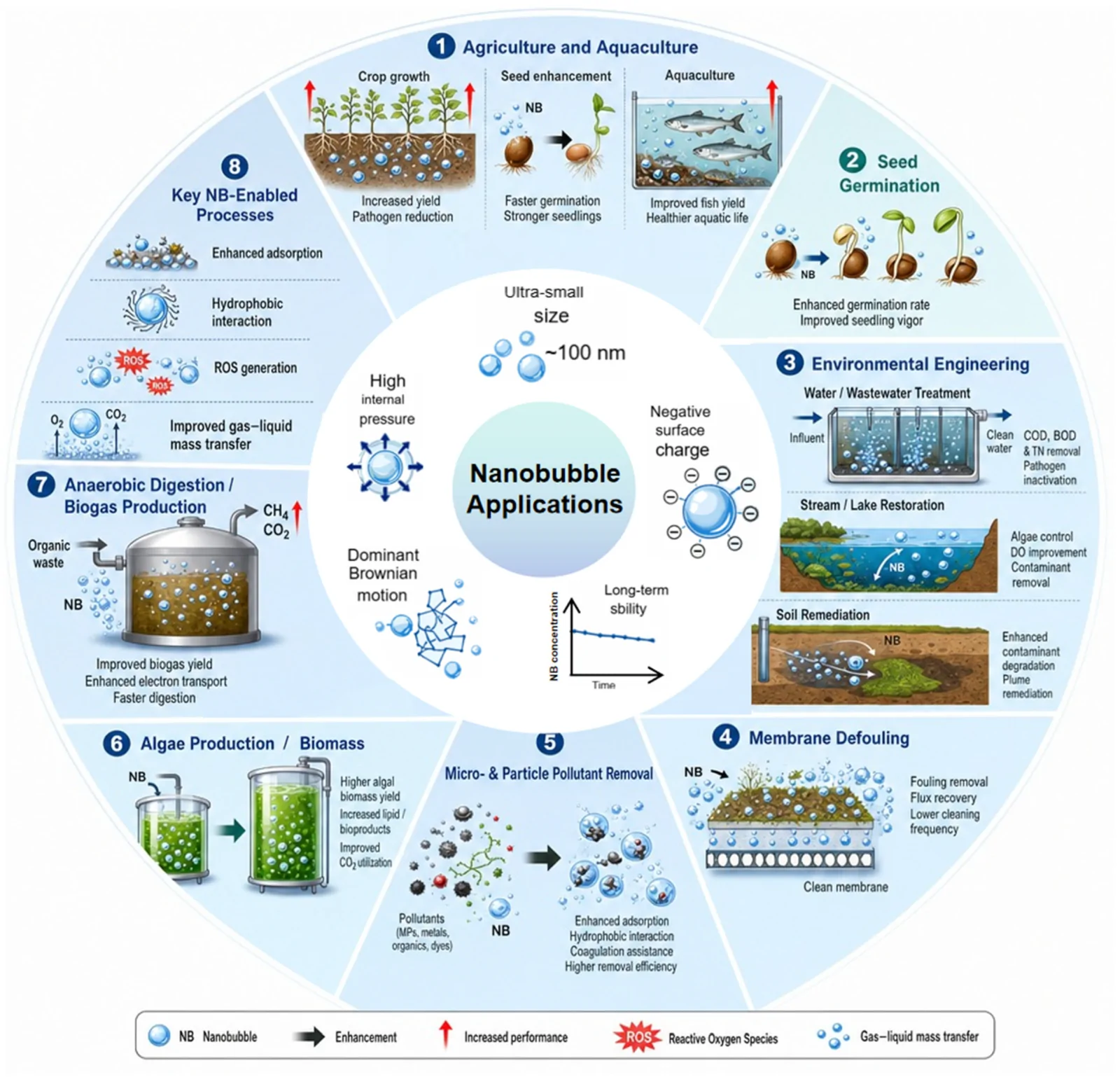
Multidisciplinary applications of the physicochemical properties of nanobubbles (NBs).

Nanobubble (NB) water technology has emerged as a transformative strategy for resolving this oxygen deficit at its source. The main physicochemical properties that determine the behavior of NBs in aqueous solutions and their main fields of application are summarized in Figure 1. NBs are characterized by an ultra-small size of approximately 100 nm, a high internal (Laplace) pressure, a negative surface charge, dominant Brownian motion, and long-term colloidal stability, with the NB concentration remaining constant over time. These properties enable NBs to remain in the irrigation water, to permeate soil pores, and to transfer gas effectively to the surrounding liquid. NBs, gas-filled cavities usually below 1 µm in diameter that can be created and maintained in an aqueous medium, provide supersaturated dissolved oxygen (DO) directly to the root system and the rhizosphere. Conventional aeration produces macro-bubbles that rise rapidly, persist only briefly, and transfer mass inefficiently, whereas NBs persist for long periods, penetrate soil pores at the microscale, and provide a long-term release of oxygen near the roots [19,20]. NB-saturated water has DO values of 15–30 mg L^−1^, which are significantly higher than those of conventionally aerated water (8–10 mg L^−1^) [21]. The one-time and spot provision of oxygen makes NB technology a scalable, non-chemical solution to overcome hypoxic injury in stress-compromised crops [20,22]. The agronomic basis of this approach was provided by Ebina et al. [23], who showed that NB-treated water improved the growth of plants, fish, and mice. Subsequent investigations have documented crop responses to NB oxygenation, including improved nutrient uptake in rice (*Oryza sativa* L.) [24], greater phosphorus availability and yield in maize (*Zea mays* L.) [25], alleviation of nanoparticle stress in wheat (*Triticum aestivum* L.) [26], and amelioration of saline-alkali soil properties and higher yield in cotton (*Gossypium hirsutum* L.) [27]. More recently, microbiome-resolved studies, such as the analysis of rhizosphere and root-endophytic bacterial communities in tomato (*Solanum lycopersicum* L.) grown in mildly saline soil under micro/nanobubble-oxygenated drip irrigation [28], have begun to resolve the mechanistic basis of NB-conferred benefits, whereas plant-level transcriptomic, proteomic, and metabolomic evidence remains scarce. The eight radiating sectors illustrate the principal application areas: (1) agriculture and aquaculture, (2) seed germination, (3) environmental engineering, (4) membrane defouling, (5) removal of micro- and particulate pollutants, (6) algae production and biomass, (7) anaerobic digestion and biogas production, and (8) key NB-enabled processes, namely enhanced adsorption, hydrophobic interactions, generation of reactive oxygen species (ROS), and improved gas–liquid mass transfer (Figure 1).

### 1.4. Novelty and Scope of This Review

Despite substantial individual progress in (i) understanding plant hypoxia signaling (ERF-VII/HRG networks), (ii) characterizing the physicochemical behavior of NBs, and (iii) expanding nano-enabled agriculture approaches, no existing review has constructed a mechanistic bridge linking NB-derived oxygen delivery to the molecular cascades of salinity- and drought-induced hypoxia, and further integrated this with nano-agricultural technologies and multi-omics decoding. The present review fills this critical gap. Specifically, we aim to: (1) establish salinity and drought as converging drivers of root-zone hypoxia and characterize the underlying plant molecular responses; (2) comprehensively describe NB water technology, its physicochemical principles, generation methods, and soil–plant-water interactions; (3) provide a mechanistic, signaling-level account of how NB-derived oxygen mitigates abiotic stress-induced hypoxia in crops; (4) integrate NB technology within the broader framework of nano-enabled agriculture; (5) map the multi-omics tools, transcriptomics, proteomics, metabolomics, epigenomics, ionomics, and phenomics) that can decode plant responses to NB water treatment; (6) discuss the applicability of the NB-oxygenation model to C3, C4, and CAM species (Section 5.6).

## 2. Abiotic Stress-Induced Root-Zone Hypoxia: Mechanisms and Consequences

### 2.1. Salinity-Induced Root-Zone Hypoxia

Salt stress (salinity) in soil causes hypoxic stress both physically and physiologically. Physically, Na^+^ ions cause soil colloids to disperse and damage the structure of soil aggregates, resulting in high soil compaction, low macroporosity, and markedly reduced gas permeability [12,16]. Saline-alkali soils have an associated high-pH environment that further increases nutrient fixation and nutrient imbalance and, in addition to an oxygen-deficient rhizosphere, leads to a compounded stress complex that negatively impacts soil function [29]. Under similar irrigation conditions, the reductions in oxygen diffusion rates in saline soils can be up to 60–80% compared to non-saline soils [12]. The high external salt levels decrease cell membrane permeability, hinder oxygen transport in the apoplast, and trigger a greater respiratory demand for O_2_ due to the activation of energy-consuming pathways for ion exclusion (SOS pathway, Na^+^/K^+^-ATPases, H^+^-ATPases) at the cellular level [11]. Under salinity, the metabolic cost of maintaining ion homeostasis also consumes ATP and increases cellular oxygen demand, creating a hypoxic environment even if bulk soil has moderate oxygen levels [11,12]. The main molecular mechanism of ionic stress response is the SOS (Salt Overly Sensitive) signaling pathway, which needs a continuous supply of ATP for Na^+^ efflux at the plasma membrane [11]. In the absence of oxygen, ATP production via oxidative phosphorylation can be restricted, which in turn can directly reduce the ability to exclude ions and lead to escalating ionic toxicity. Salt tolerance is further improved by SOS2, which inhibits CTR1 through the mechanism of phosphorylation and turns on responses in the ethylene signaling pathway that in turn converge on oxygen-sensing pathways [10]. This SOS–ethylene–O_2_ cross-talk reveals the mechanisms of stress amplification in response to salinity-induced hypoxia.

### 2.2. Drought-Induced Root-Zone Hypoxia

Drought causes hypoxia in the root zone in ways similar to those of salinity but not identical. When soil moisture is declining, the soil dries quickly, which results in compaction of the soil particles, decreased pore continuity, and collapse of the macropore network through which gaseous oxygen diffuses, especially in clayey soils [15]. Even if the liquid water content in the rhizosphere seems to be sufficient for root activity, the progressive decline of gas-phase diffusion restricts O_2_ delivery [14]. From a physiological perspective, drought causes stomatal closure (mainly triggered by ABA), which decreases CO_2_ fixation and the export of photosynthate to roots. Root respiration, which relies on photosynthate-derived substrates, is thus constrained, while simultaneously the demand for ATP through osmotic adjustment processes (proline biosynthesis, compatible solute accumulation) remains high [8]. This metabolic imbalance, in which O_2_ demand exceeds supply, arises in the intracellular space under water-limited conditions even when the soil is not flooded [14]. ABA signaling and the regulation of auxin biosynthesis and transport also seem to contribute to changes in root architecture, such as decreased lateral root density and impaired root hair development, which further restrict the root surface area available for the uptake of O_2_ [30]. Aquaporin channels (plasma-membrane intrinsic proteins, PIPs; tonoplast intrinsic proteins, TIPs), whose expression is reduced under drought stress through ABA-dependent signaling, are water and gas transporters. They are downregulated during drought, which decreases CO_2_ and O_2_ diffusion across root cell membranes and thus adds to the depletion of O_2_ in the cell [9,30].

### 2.3. Combined Salinity-Drought Stress: Compounded Hypoxia

Salinity stress and drought stress interact with each other, and this combination of the two is projected to be more common in future climate scenarios, giving rise to an additional hypoxic stress scenario that includes both physical factors of lowering soil oxygen (caused by compaction induced by salinity and dry soil reducing oxygen gas availability) and the physiological factors of increased oxygen demand (caused by simultaneous osmotic stress and ionic stress response). When water is present, salinity decreases the osmotic water potential of the soil solution, causing a physiological drought, whereas water deficiency directly decreases the soil water potential [9]. The dual impairment of water uptake triggers rapid stomatal limitation, restricts CO_2_ assimilation, and predisposes the photosynthetic apparatus to excess excitation energy and electron leakage, enhancing ROS formation in chloroplasts and mitochondria [8]. Barrett-Lennard [12] documented that the interaction between waterlogging and salinity produces physiological effects more severe than either stress alone, noting a particularly damaging compounding of hypoxia-driven root metabolic dysfunction and salt-induced membrane injury. The combined stress also impairs aerenchyma formation, a key hypoxia-adaptive structure, because ethylene signaling required for lysigenous aerenchyma development is disrupted by ionic toxicity [15]. Furthermore, as demonstrated by Zandalinas et al. [8], the combination of drought and high temperature (frequently co-occurring with drought) further depletes root-zone antioxidant capacity, making recovery from co-existing hypoxic episodes markedly slower.

### 2.4. Molecular Consequences of Root-Zone Hypoxia in Crops

#### 2.4.1. Shift from Aerobic Respiration to Fermentative Catabolism

The main cellular response to oxygen deficiency is a change in metabolism from the very efficient oxidative phosphorylation pathway to oxygen-independent glycolysis and ethanolic fermentation. The production of ATP molecules by aerobic respiration is about 36 per molecule of glucose, but only two molecules of ATP are produced by fermentation, causing a rapid and severe energy problem in hypoxic root cells [18]. Pyruvate is formed during glycolysis, which under hypoxic conditions can be converted to acetaldehyde by pyruvate decarboxylase (PDC) before being degraded to ethanol by alcohol dehydrogenase (ADH), or to lactate by lactate dehydrogenase (LDH) [18]. Accumulation of ethanol and acetaldehyde is highly phytotoxic, as both compounds disrupt membrane integrity, denature proteins, and inhibit normal cellular metabolism [18]. Under low oxygen conditions, the induction of ADH activity has been observed in several plant species such as wheat [13], rice [31], and maize [32]; ADH activity is a widely used enzymatic marker for the severity of hypoxia in root tissue. Lactate accumulation, caused by LDH, is tolerated at first but leads to cytoplasmic acidosis, a progressive decrease in the intracellular pH, which causes enzyme dysfunction, interferes with ion transport, and ultimately leads to cell death.

#### 2.4.2. ERF-VII/HRG Oxygen-Sensing Cascade

**Figure 2:**
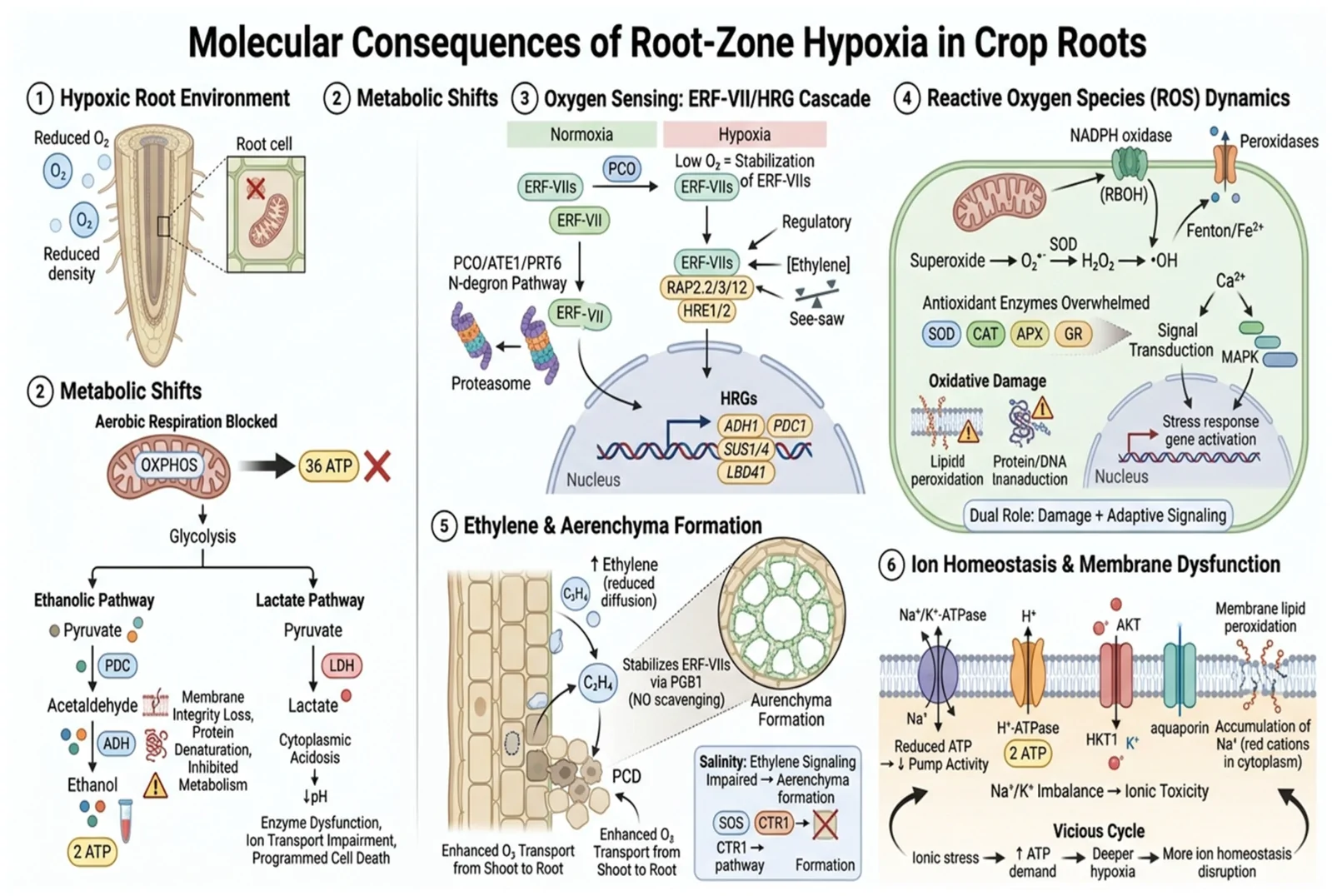
Schematic overview of the molecular consequences of root-zone hypoxia in crops. The colored circles represent different molecular species, metabolites, and ions involved in hypoxia-induced physiological responses, whereas red cross symbols (✕) indicate inhibition or impairment of the corresponding biological processes. Upward (↑) and downward (↓) arrows denote increasing and decreasing trends in the respective traits.

Plants have a complex molecular oxygen-sensing system that is based on the N-degron proteolytic pathway. In *Arabidopsis thaliana* (L.) Heynh., plant cysteine oxidase (PCO) enzymes bind to and oxidize the N-terminal cysteine residue of Group VII Ethylene Response Factors (ERF-VIIs), such as RAP2.2, RAP2.3, RAP2.12, HRE1 and HRE2, for eventual proteasomal degradation through the arginyl-tRNA-protein transferase (ATE1/ATE2) and the N-recognin PRT6 ubiquitin ligase complex [33]. Hypoxia reduces the activity of the PCO in favor of the stabilization of ERF-VIIs, which then enter the nucleus, thus leading to the transcription of hypoxia-responsive genes (HRGs) such as ADH1, PDC1, SUS1/4 and LBD41 [33]. This transcriptional reprogramming re-routes carbon metabolism towards anaerobic energy production and activates ROS detoxification pathways, aerenchyma formation, and adventitious root development to promote access to oxygen [34]. Nitric oxide (NO) generated under hypoxia further modulates ERF-VII stability; NO-dependent reactions can suppress or promote ERF-VII activity depending on concentration and cellular context, integrating nitrogen status with oxygen sensing [35]. The cross-talk between oxygen concentration, NO, and ethylene at the ERF-VII regulatory node makes this cascade a prime target for NB-mediated intervention (Figure 2).

#### 2.4.3. Reactive Oxygen Species Dynamics Under Hypoxia

Paradoxically, while hypoxia is defined by oxygen insufficiency, it is also associated with elevated ROS production through mitochondrial electron transport chain dysfunction, NADPH oxidase (RBOH) activation, and peroxidase activity [35]. The incomplete reduction of O_2_ at dysfunctional mitochondrial complexes I and III generates superoxide (O_2_•^−^), which is dismutated to hydrogen peroxide (H_2_O_2_) by superoxide dismutases (SODs). H_2_O_2_, in turn, drives hydroxyl radical (•OH) formation through Fenton chemistry when free iron is available, a situation common in hypoxic, anaerobically metabolizing root cells [35,36]. Prolonged exposure to hypoxia causes an overload of the antioxidant defense system (superoxide dismutase (SOD), catalase (CAT), ascorbate peroxidase (APX), and glutathione reductase (GR)), which results in oxidative damage to the membrane lipids, proteins, and nucleic acids [13]. ROSs also act as signaling molecules during hypoxia, triggering an influx of calcium, MAPK cascades, and stress-induced gene expression, having a dual role as damage mediators and adaptive signals [35]. The dual ROS function has significance for NB treatment, as the bubble collapse-induced production of •OH could be a priming response rather than a damaging response.

#### 2.4.4. Ethylene Accumulation and Aerenchyma Induction

The gaseous phytohormone ethylene (C_2_H_4_) is a central regulator of hypoxia responses. Under oxygen deficiency, reduced diffusion of ethylene from plant tissues leads to its rapid accumulation, particularly in submerged or compaction-affected roots [15]. Ethylene acts indirectly on ERF-VIIs by stimulating the expression of phytoglobin 1 (PGB1), which scavenges NO and thus inhibits the degradation of ERF-VIIs [33]. Aerenchyma also forms through programmed cell death in the root cortex, creating gas-filled channels that allow O_2_ to move from the above-ground tissues to the hypoxic roots; this occurs at the onset of ethylene production [15]. Under salinity stress, however, the ionic toxicity impairs normal ethylene perception, disrupting aerenchyma formation and leaving roots without this key adaptive mechanism [12]. The interaction between the SOS pathway and CTR1, the ethylene signaling suppressor, further complicates ethylene-mediated hypoxia responses under salinity [10].

#### 2.4.5. Ion Homeostasis Disruption and Membrane Dysfunction

The activity of energy-dependent ion transporters is directly affected by a decrease in energy availability in the roots in response to hypoxia. Under saline conditions, the Na^+^/K^+^-ATPases and H^+^-ATPases, which depend on constant ATP for their activity to maintain the ionic gradient across the cell membrane, become functionally impaired; as a result, Na^+^ accumulates to toxic levels inside the cell while K^+^ leaks out [11]. At the same time, ROS generated by hypoxia cause lipid peroxidation of the cell membranes, which in turn leads to loss of structural integrity of the plasma membranes and impairs the function of ion channel proteins, such as HKT1 Na^+^ transporters and AKT K^+^ channels, and aquaporin water channels [32]. The combined energetic and structural membrane impairment under hypoxia thus creates a vicious cycle: ionic stress worsens, which further increases metabolic demand for ATP, which deepens hypoxia, which further disables energy-dependent ion homeostasis.

Figure 2 summarizes in a single schematic the five molecular consequences of root-zone hypoxia. Panels 1 and 2 show the hypoxic root environment, in which reduced O_2_ blocks oxidative phosphorylation (OXPHOS) and shifts metabolism from the efficient aerobic pathway (about 36 ATP per glucose) to ethanolic and lactate fermentation (2 ATP per glucose), with accumulation of acetaldehyde, ethanol, and lactate and the associated membrane damage, protein denaturation, cytoplasmic acidosis, and cell death. Panel 3 depicts the ERF-VII/HRG oxygen-sensing cascade: under normoxia, ERF-VII transcription factors are destabilized through the PCO/ATE1/PRT6 N-degron pathway, whereas under hypoxia they are stabilized, enter the nucleus, and activate hypoxia-responsive genes (HRGs) such as ADH1, PDC1, SUS1/4, and LBD41. Panel 4 shows ROS dynamics, including superoxide and H_2_O_2_ production, Fenton chemistry, lipid peroxidation, and the dual role of ROS as damage mediators and as signals acting through Ca^2+^ and MAPK cascades. Panel 5 shows ethylene accumulation and the induction of aerenchyma by programmed cell death, and indicates that salinity impairs ethylene signaling through the SOS–CTR1 pathway. Panel 6 shows the vicious cycle of ion homeostasis and membrane dysfunction, in which a reduced ATP supply compromises Na^+^/K^+^-ATPases, H^+^-ATPases, HKT1, AKT channels, and aquaporins, so that ionic stress increases the ATP demand and further deepens hypoxia.

## 3. Nanobubble Water Technology: Physics, Chemistry, and Agricultural Properties

### 3.1. Classification and Physicochemical Characteristics

**Figure 3:**
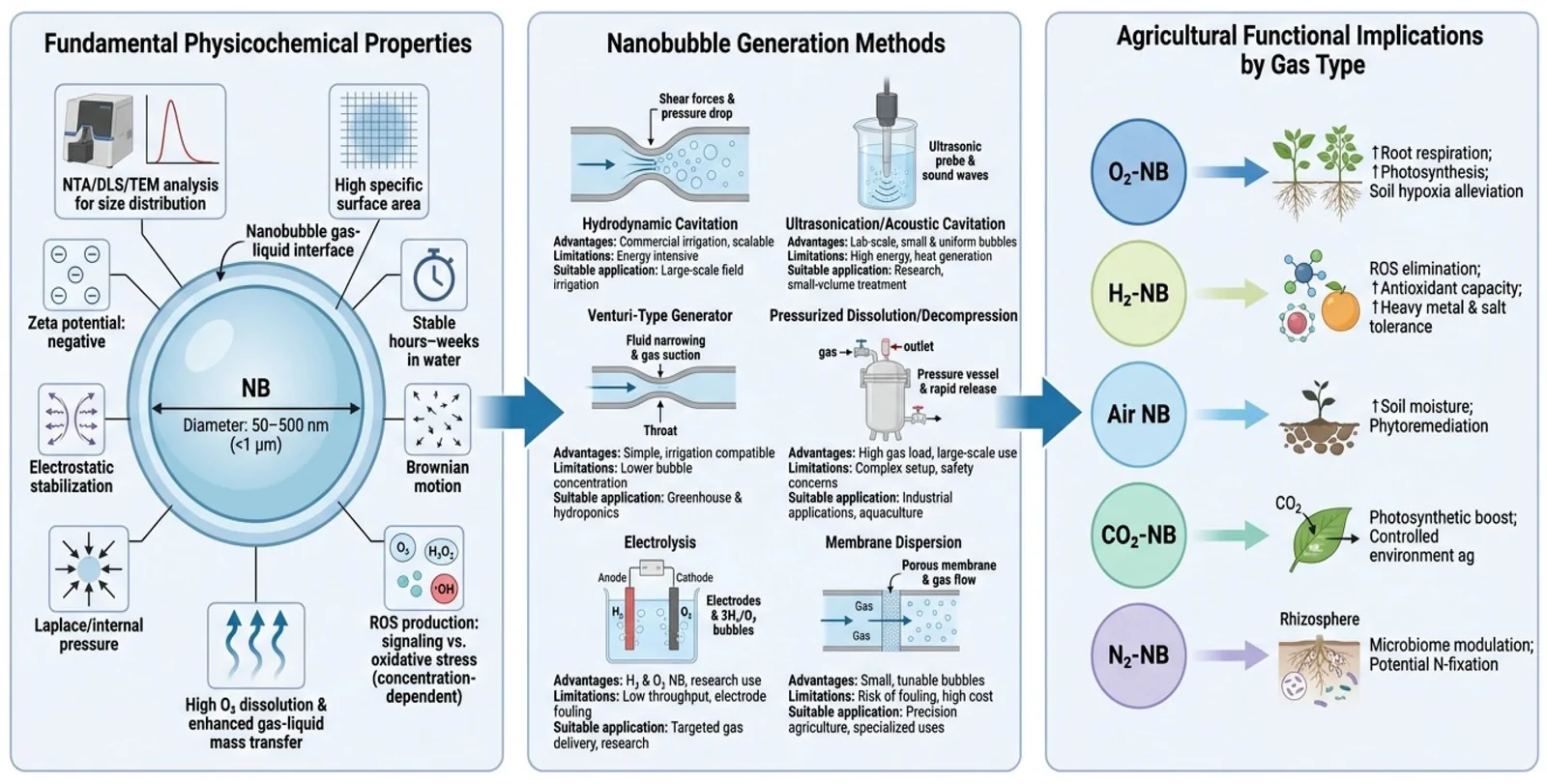
Physicochemical properties of nanobubbles, generation techniques, and functional implications in agriculture.

Gaseous micro- and nanobubbles (MNBs) can be classified on the basis of their diameter: microbubbles (10–100 µm), submicron bubbles (1–10 µm), and nanobubbles (<1 µm). High internal pressure: The internal gas pressure of a bubble is inversely proportional to its radius (Young–Laplace equation) [20,22], making nanobubbles particularly useful for delivering oxygen to agricultural crops. The internal pressures of nanobubbles are orders of magnitude greater than those of macro-bubbles and are thought to enhance gas dissolution into the surrounding liquid phase [19]. Exceptional stability: Unlike macro-bubbles, which rise and burst within seconds, NBs have minimal buoyancy and remain highly stable in aqueous suspension for hours or days without significant dissolution [19,20]. This stability is thought to be due to the buildup of negative surface charges (zeta potential of approximately −30 to −50 mV) that create electrostatic repulsion between adjacent bubbles and inhibit coalescence. High surface-area-to-volume ratio: NBs are produced at concentrations of 10^6^–10^8^ per mL, providing a collectively enormous gas–liquid interfacial area for mass transfer of O_2_ into the aqueous phase and subsequently into plant root cells [22]. ROS generation: Upon collapse under physical stimuli (acoustic, hydrodynamic), NBs generate hydroxyl radicals (•OH) through localized cavitation energy release, a phenomenon with documented signaling and antimicrobial implications in agricultural systems [36,37]. Surface tension reduction: High concentrations of negatively charged NBs reduce the surface tension of water, improving infiltration through soils and substrates and enhancing capillary action for water and dissolved nutrient delivery [38] (Figure 3).

### 3.2. Generation Technologies for Agricultural Applications

Hydrodynamic cavitation: The method uses high-velocity water in a specially designed nozzle or orifice, which induces local pressure drops, nucleating and collapsing gas bubbles to produce NBs through these dynamics. This is an energy-efficient solution and can be scaled to field-level irrigation systems [20]. Acoustic (ultrasonic) cavitation: Alternating pressure waves are created in liquid by high-frequency ultrasound, which results in cavitation events and submicron bubbles. Although it is a good method for generating NBs in the lab, the energy costs are a constraint for field-scale production [22]. Direct electrolysis: Water is electrolyzed to produce H_2_ and O_2_ NBs at electrode surfaces. Electrolytic production of oxygen-NB water gives very high DO concentrations and is increasingly considered for precision irrigation [20]. Membrane diffusion: Pressurized gas (O_2_ or air) is passed through a hydrophobic microporous membrane that is in contact with water, creating fine bubbles at the surface of the membrane. It is possible to control the size and composition of the bubbles with this method [19]. Pressure dissolution: Under high pressure, gas is dissolved in water, and when the pressure is released, nano- and micro-scale bubbles nucleate in the water volume. This is the working principle of commercial NB irrigation systems that are extensively used in controlled-environment agriculture. The generation methods and their advantages and limitations are compared in Figure 3.

### 3.3. Dissolved Oxygen Dynamics in NB Irrigation Water

**Figure 4:**
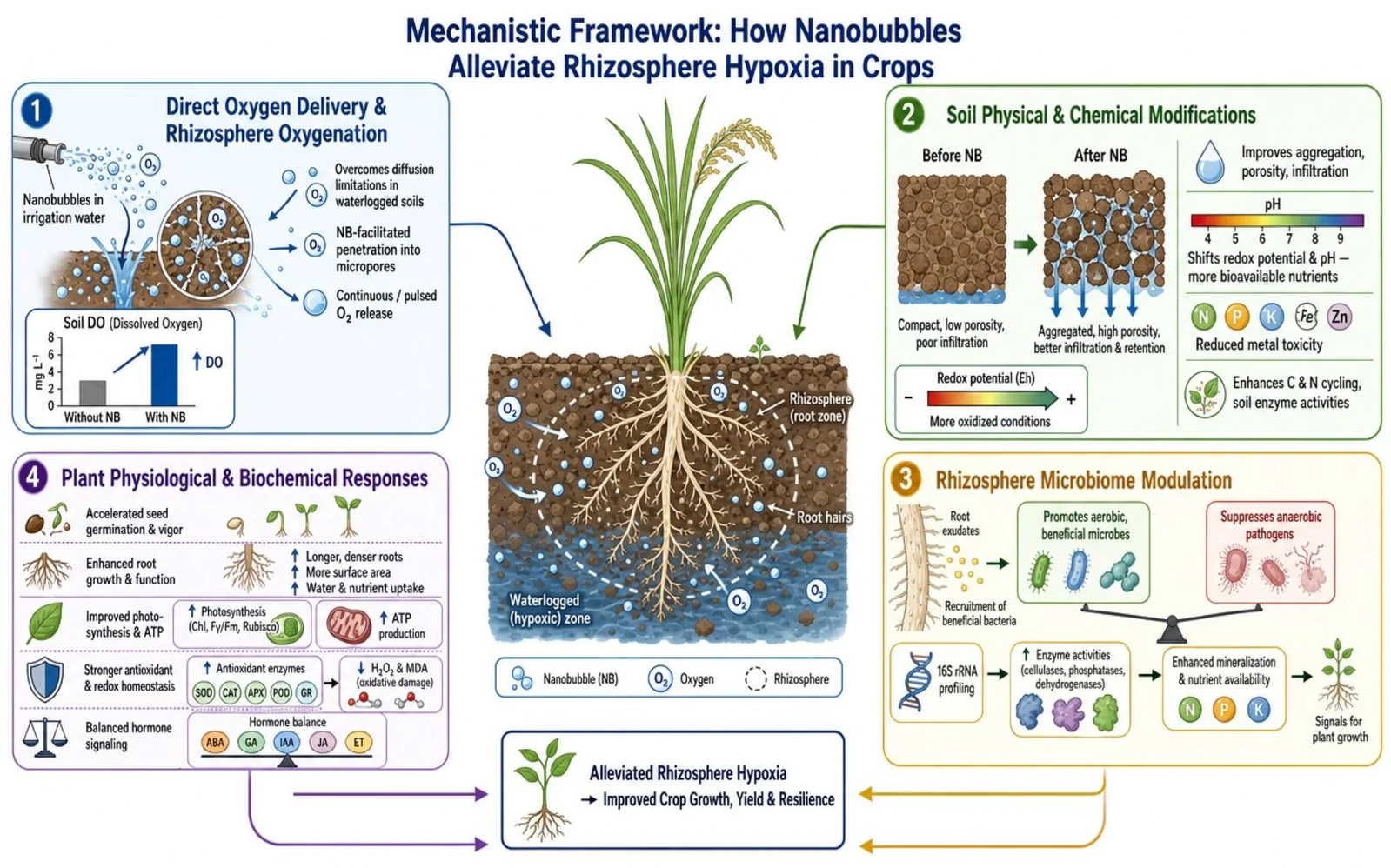
Mechanistic framework of nanobubble-mediated alleviation of rhizosphere hypoxia in plants. Upward (↑) and downward (↓) arrows denote increasing and decreasing trends in the respective traits.

One of the most agriculturally significant properties of NB water is its capacity to carry supersaturated DO concentrations with exceptional temporal stability. While conventional aeration achieves DO of 8–10 mg L^−1^ near ambient air saturation, oxygen-NB water routinely achieves DO concentrations of 15–30 mg L^−1^ depending on generation method and temperature [21,25]. Critically, the elevated DO in NB water persists throughout irrigation delivery systems at distances and time intervals impractical for conventional aeration, a consequence of the resistance to bubble dissolution conferred by NB physicochemical stability [19,20]. The high DO content of NB irrigation water has been demonstrated to penetrate into soil pore networks and diffuse into the rhizosphere, elevating rhizospheric DO concentrations well above values measured under conventional irrigation [17]. MNBs in the root-zone environment can penetrate soil pores and reach the rhizosphere directly due to their nanoscale diameter and Brownian motion; produced at concentrations of 10^6^–10^8^ mL^−1^, they disperse widely and penetrate confined or hard-to-reach spaces more effectively than macro-bubbles [22,39]. Direct oxygen delivery and rhizosphere oxygenation show that NBs in irrigation water overcome diffusion limitations in waterlogged or compacted soils, penetrate soil micropores, and release oxygen continuously or in pulses, which raises soil dissolved oxygen (DO) compared with untreated soil (Figure 4).

### 3.4. NB Water Effects on Soil Physicochemistry

Beyond DO delivery, NB irrigation modifies key soil physicochemical properties relevant to abiotic stress management. MNB irrigation with treated or oxygenated water has been demonstrated to significantly reduce soil electrical conductivity (EC) stress in the root zone: moderate DO concentrations (15 mg L^−1^) lowered EC by 63–73%, whereas high DO concentrations (30 mg L^−1^) resulted in reductions of 60–92% compared to non-cultivated saline controls [21]. The mechanisms include: (i) enhanced water infiltration through reduced surface tension, facilitating salt leaching from the root zone; (ii) improved soil aggregate stability through microbial activity stimulated by elevated DO; and (iii) a possible displacement of adsorbed Na^+^ from soil colloids by migrating NBs, which remains to be tested experimentally. NB-driven soil oxygenation also modifies soil pH through higher aerobic microbial respiration that generates organic acids and CO_2_, relevant to saline-alkali soil management, where high pH limits nutrient availability. Soil physical and chemical modification shows that NB treatment produces a more aggregated and porous soil with better infiltration and water retention, a higher redox potential (more oxidized conditions), a more favorable pH, reduced metal toxicity, improved carbon and nitrogen cycling, and higher soil enzyme activity. MNBs alone reduced soil pH by approximately 5% in saline-alkali contexts [27,40]. Additionally, NB irrigation has been shown to enhance soil phosphorus availability through stimulation of alkaline phosphatase-secreting rhizobacteria [25]. Rhizosphere microbiome modulation indicates that NBs promote aerobic beneficial microbes and suppress anaerobic pathogens, a shift that is typically resolved by 16S rRNA profiling, and that these changes stimulate enzyme activities and nutrient mineralization and may generate plant-growth-promoting signals (Figure 4). Soil aeration may also reduce greenhouse gas emissions because aerobic conditions favor N_2_ rather than N_2_O during denitrification [41]; for example, air injection into paddy soil, a process analogous to NB oxygenation, reduced N_2_O emissions by 17% and NH_3_ volatilization by 16% while increasing nitrogen uptake by 8.3% [42]. Plant physiological and biochemical responses include accelerated germination and vigor, enhanced root growth and surface area, greater water and nutrient uptake, improved photosynthesis and ATP production, balanced hormone signaling (ABA, ethylene, GAs, and JA), and a stronger antioxidant defense with reduced oxidative damage (lower H_2_O_2_ and malondialdehyde). Together, these modules reduce rhizosphere hypoxia and improve crop growth, yield, and resilience [23] (Figure 4).

## 4. Mechanistic Pathways Through Which Nanobubble Oxygen Mitigates Hypoxia-Induced Damage

### 4.1. Restoration of Aerobic Respiration and ATP Homeostasis

The most fundamental and immediate effect of NB-derived oxygen delivery to stressed root systems is the restoration of mitochondrial aerobic respiration. Elevated rhizospheric DO, driven by NB-dissolved oxygen, replenishes the terminal electron acceptor supply for the mitochondrial electron transport chain (ETC), enabling re-engagement of Complexes I-IV and ATP synthase for oxidative phosphorylation [18]. The shift from fermentative ATP yield (2 ATP/glucose) back to oxidative phosphorylation (approximately 36 ATP/glucose) represents an approximately 18-fold increase in energy generation efficiency, rapidly replenishing the cellular ATP pool. In wheat, nanobubble irrigation improved plant growth, nutrient status, photosynthesis, and antioxidant capacity [26], responses that are consistent with an improved root energy status, although direct measurements of root ATP pools under NB treatment are still needed. The restored ATP pool directly powers energy-dependent processes impaired by hypoxia: Na^+^/K^+^-ATPases resume Na^+^ exclusion from the cytoplasm, H^+^-ATPases re-establish plasma-membrane proton gradients critical for secondary active transport of minerals, and aquaporin gating proteins resume their molecular trafficking functions [11].

### 4.2. Suppression of ERF-VII/HRG Signaling and Fermentative Gene Expression

The effect of NB-derived oxygen restoration and its mechanistic link with molecular hypoxia signaling seems to be mediated mainly by the ERF-VII/N-degron pathway. The increasing levels of O_2_ supplied by NBs restore cellular O_2_ levels to above the critical level, thereby reactivating the PCO enzyme that catalyzes the oxidation of the N-terminal Cys residue of ERF-VIIs. Recognized by ATE1/ATE2, oxidized ERF-VIIs are degraded by the PRT6/UPS complex, which eliminates their transcriptional activation of HRGs [33]. This ERF-VII suppression under restored oxygen conditions directly downregulates the expression of fermentation-pathway genes (ADH1, PDC1, LDH), reducing the production of phytotoxic ethanol and acetaldehyde, a pivotal step in reversing hypoxic injury [18]. While direct molecular evidence for NB-specific ERF-VII suppression in crop plants awaits transcriptomic confirmation, parallel evidence from oxygen nanobubble studies in tumor hypoxia models has consistently demonstrated that NB-mediated oxygen delivery reduces HIF-1α expression, the functional animal analog of plant ERF-VII, by 75% at the mRNA level and 25% at the protein level, reversing cellular hypoxic transcriptional programs [43]. The structural and functional parallels between HIF-1α (animals) and ERF-VII (plants) as oxygen-sensing transcription factors stabilized under hypoxia suggest mechanistically similar NB responsiveness in plant systems.

### 4.3. Rebalancing Ion Homeostasis Under Salinity Stress

One of the major advantages of NB oxygen irrigation under saline conditions is the restoration of ion homeostasis in root cells. Experimental data from saline-soil studies are supportive: micro/nanobubble-oxygenated drip irrigation increased tomato yield in mildly saline soil and was correlated with changes in rhizosphere and root-endophytic bacterial populations [28], while micro-nanobubble oxygenation improved soil properties, cotton physiology, and yield under saline conditions [27]. Direct measurements of root Na^+^/K^+^ ratios and plasma-membrane transporter activities under NB treatment are rare. The proposed mechanism is an ATP-dependent reactivation of both Na^+^/K^+^-ATPases and H^+^-ATPases, driven by NB-reactivated oxidative phosphorylation, leading to increased Na^+^ extrusion by SOS1 Na^+^/H^+^ antiporters and K^+^ retention through the regulation of high-affinity K^+^ transporters (HKT) [11]. NB irrigation has also been shown to lower the EC of the soil at the root surface by 60–92% at high DO concentrations [21], thus lowering the ionic load on root cells. This dual action, in which NB irrigation both restores ATP supply for cellular ion homeostasis and lowers soil electrical conductivity (EC) by reducing the ionic burden, makes NB irrigation particularly effective as a comprehensive ionic stress management strategy. Fungal community assembly and root–fungal interactions have also been found to mediate water–oxygen coupling effects under nanobubble-oxygenated drip irrigation in tomato grown in saline soil [44], supporting the role of the rhizosphere microbiome in NB-mediated salt tolerance. A combined transcriptomic, proteomic, and metabolomic study of the regulation of ion transport by NB has yet to be carried out.

### 4.4. ROS Dynamics: Detoxification and Signaling Priming Under NB Treatment

The interaction between NB technology and cellular ROS metabolism operates on two levels. First, the restoration of aerobic respiration under NB-supplied oxygen normalizes mitochondrial electron flow, reducing the electron leakage that generates O_2_•^−^ and H_2_O_2_ under hypoxia-induced mitochondrial dysfunction [35]. This normalization effectively reduces the oxidative load on the cell, allowing the constitutive antioxidant system (SOD, CAT, APX, GR) to scavenge residual ROS from the stress period. Second, the •OH radicals generated during NB collapse, while potentially damaging at high concentrations, appear at subthreshold concentrations to function as redox priming signals that pre-condition the antioxidant defense system [36,37]. Liu et al. [37] revealed that NBs generate ROS, which can be beneficial to seed germination in small amounts but harmful in large quantities. This implies that subthreshold NB-induced ROS can prime the antioxidant network to better cope with ROS during a subsequent stress event; however, there is limited direct evidence for stressed crops. Micro-nanobubble oxygenation activates micro-nanobubbles in the rhizosphere soil, which in turn stimulates the microbial community, accelerating organic matter decomposition and releasing metabolites that optimize soil structure [27]. A dual-level ROS modulation by NBs, which includes reducing ROS overproduction under chronic hypoxia and priming the antioxidant system through controlled release of •OH, is a sophisticated redox management strategy with significant implications for salinity and drought tolerance.

### 4.5. Aquaporin Regulation and Root Hydraulic Conductivity Recovery

Aquaporins, transmembrane channel proteins that facilitate the movement of water, CO_2_, O_2_, and other small molecules across cellular membranes, are critical mediators of root hydraulic conductivity and gas transport in root cells [9]. Their expression and gating activity are reduced under both drought (via ABA-induced phosphorylation-dependent channel closure) and hypoxia (via ROS-induced S-nitrosylation and H_2_O_2_-mediated gating inhibition) [9,30]. The resulting impairment of aquaporin function compounds the restriction of water and gas transport in stressed roots. In alfalfa, micro-nanobubble oxygenation under subsurface drip irrigation improved soil enzyme activities, microbial abundance, and yield, and structural equation modeling identified the rhizosphere mechanisms involved [14]. To our knowledge, however, direct evidence that NB treatment regulates aquaporin expression or root hydraulic conductivity in crops has not yet been reported. The restoration of cellular redox balance under NB-delivered oxygen, removing the H_2_O_2_- and NO-mediated aquaporin gating inhibition, is a plausible but still untested mechanism through which NB treatment recovers aquaporin-mediated water and gas transport in stressed root systems.

### 4.6. Ethylene and Hormonal Cross-Talk Modulation

As NB-delivered oxygen normalizes cellular O_2_ concentrations, the ethylene accumulation characterizing hypoxic roots is addressed through dual mechanisms. First, restored aerobic conditions facilitate enzymatic ethylene degradation and normal tissue diffusion, reducing ethylene-driven ERF-VII stabilization and HRG transcription [33]. Second, improved root energy status under NB oxygenation may re-engage ABA signaling pathways, particularly the ABA-mediated stomatal regulation that coordinates above-ground water use with root-zone oxygen status [9]. The ABA-ethylene-O_2_ regulatory triangle is particularly important under drought stress, where ABA-driven stomatal closure reduces transpiration but also limits the photosynthate supply to roots that supports aerobic respiration. NB irrigation, by providing an exogenous O_2_ source independent of photosynthate supply, may partially decouple root aerobic metabolism from the ABA-driven constraints on photosynthesis during drought, providing roots with energetic independence during the critical water-deficit period. This partial metabolic autonomy of roots under NB irrigation may explain the improved root hydraulic conductivity and sustained root growth observed under NB treatment during drought stress [14].

### 4.7. Rhizosphere Microbiome Restoration and Plant-Growth-Promoting Effects

NB oxygen delivery exerts profound effects on the rhizosphere microbial community, whose diversity and activity are severely impaired under hypoxic, saline, and drought conditions. Obligate anaerobic bacteria that proliferate under hypoxic rhizosphere conditions, including potential plant pathogens and nitrate-reducing organisms, are suppressed by NB-elevated DO, while aerobic plant-growth-promoting rhizobacteria (PGPR) are selectively enriched [25,45]. Both oxygen and nitrogen nanobubble types significantly alter rhizosphere microbial community composition; high-concentration NB treatments increase the relative abundance of multiple beneficial rhizobacteria involved in nutrient cycling, including Bacteroidota, Chitinophagales, Gemmatimonadota, and *Ureibacillus*, with enhanced microbial connectivity and reduced niche specialization in NB-treated soils [45]. Micro-/nanobubble oxygenation irrigation altered the structure and diversity of key inter-root microbial communities in maize and facilitated the secretion of alkaline phosphatases, which increased soil phosphorus effectiveness and enhanced crop yield [25]. The restoration of aerobic microbial populations under NB irrigation also activates nitrogen cycling pathways: NB oxygenation has been associated with enhanced nitrification and plant-available N release, improving plant nitrogen availability at a time when energy-intensive N uptake mechanisms are compromised by hypoxia [46,47].

## 5. Crop-Specific Evidence: NB Water Under Salinity and Drought Conditions

### 5.1. Rice (Oryza sativa)

Rice, a globally critical staple crop grown predominantly in submerged conditions, presents a unique context for NB water research; it must balance the hypoxia-tolerant flooding physiology used for paddy cultivation with emerging aerobic or water-saving cultivation systems where root-zone oxygen dynamics are increasingly important [32]. Wang et al. [24] showed that NBs promoted nutrient utilization and growth in rice by upregulating nutrient-uptake genes and stimulating growth-hormone production, achieving equal yield with approximately 25% less fertilizer in laboratory and field experiments. Salt-tolerant rice responded to micro-/nanobubble oxygenation under NaCl stress with improved growth and reduced lipid peroxidation, as summarized in [48], and air, oxygen, and nitrogen NBs differentially mitigated cadmium toxicity and accumulation in rice seedlings [49]. Transcriptome comparisons of lowland and upland rice under root-zone hypoxia [31] and of rice germinating under oxygen deficiency [34] provide reference sets of hypoxia-responsive genes, such as ADH1 and PDC1, that can serve as benchmarks for future transcriptomic studies on NB-mediated suppression of the hypoxic response. Integrated transcriptomic, proteomic, and metabolomic datasets for NB-treated rice under combined salt–hypoxia stress has not yet been reported. NB irrigation has also shown promise in reducing arsenic mobilization in rice paddies: oxygen NBs affect rhizosphere redox potential, reducing As (III) dissolution and decreasing arsenic uptake by rice roots [50], an important co-benefit in regions where arsenic contamination co-occurs with salinity or drought stress. Additional crop-level evidence is summarized in Table 1.

### 5.2. Maize (Zea mays)

Maize, the world’s most widely cultivated cereal, is highly sensitive to root-zone oxygen deficiency, with significant yield losses documented under waterlogging [32]. Bian et al. [25] evaluated micro-/nanobubble oxygenation irrigation in maize and found that, compared with conventional irrigation, it increased rhizosphere available phosphorus by 21.3% and alkaline phosphatase activity by 15.4%, primarily by regulating bacterial community diversity, which provides field-relevant evidence for the agronomic benefit of NB oxygenation beyond laboratory conditions. Evidence under drought or salinity in maize, including effects on aquaporin expression, ABA signaling, and antioxidant enzymes, is still lacking, and the corresponding responses described for other crops in Section 4 should therefore be regarded as hypotheses for this species. In alfalfa (*Medicago sativa* L.), a forage crop, micro-nanobubble oxygenation under subsurface drip irrigation improved soil enzyme activities, microbial abundance, and yield [14].

### 5.3. Wheat (Triticum aestivum)

Wheat root systems under salinity-hypoxia co-stress show characteristic activation of ADH and LDH fermentation pathways, even in the absence of waterlogging, confirming the physiological reality of salt-induced hypoxia documented by Barrett-Lennard [12]. Kononenko et al. [13] confirmed that wheat genotypes under combined salt and hypoxia stress exhibit elevated ADH activity, ROS accumulation, and reduced aerenchyma formation compared to stress-specific treatments, demonstrating the compounding nature of co-stress hypoxia. Zhang et al. [26] showed that nanobubble watering improved the growth and nutrient status of wheat seedlings exposed to ZnO nanoparticles (ZnO-NPs, 500 mg L^−1^), as evidenced by higher foliar nitrogen and phosphorus, and further enhanced photosynthesis and antioxidant capacity under ZnO-NP stress, indicating that NBs can alleviate nanoparticle phytotoxicity.

### 5.4. Cotton (Gossypium hirsutum)

Cotton cultivation in saline-alkali soils, particularly in arid and semi-arid regions such as Xinjiang, China, presents a critical application context for NB oxygenation irrigation. Although cotton is moderately salt-tolerant, its growth and productivity under saline-alkali conditions are substantially constrained by root-zone oxygen deficiency and soil structural degradation [27]. Yang et al. [27] conducted a comprehensive field experiment demonstrating that micro-nanobubble oxygenation irrigation effectively ameliorated saline-alkali soil properties in cotton cultivation, improving soil aeration, water retention capacity, root respiration, and antioxidant enzyme activities while significantly enhancing microbial activity in the rhizosphere. Cotton yield and fiber quality metrics improved significantly under NB irrigation at all tested salinity levels, with the greatest relative improvements observed at moderate rather than high salinity, suggesting NB oxygenation is most effective when restoring hypoxia rather than overcoming full ionic toxicity. Bian et al. [40] combined micro-/nanobubble oxygenation irrigation with microbial agents in saline soil and reported lower soil salinity, higher bacterial richness, and up to 44.53% higher seed cotton yield than the control. 

**Figure 5:**
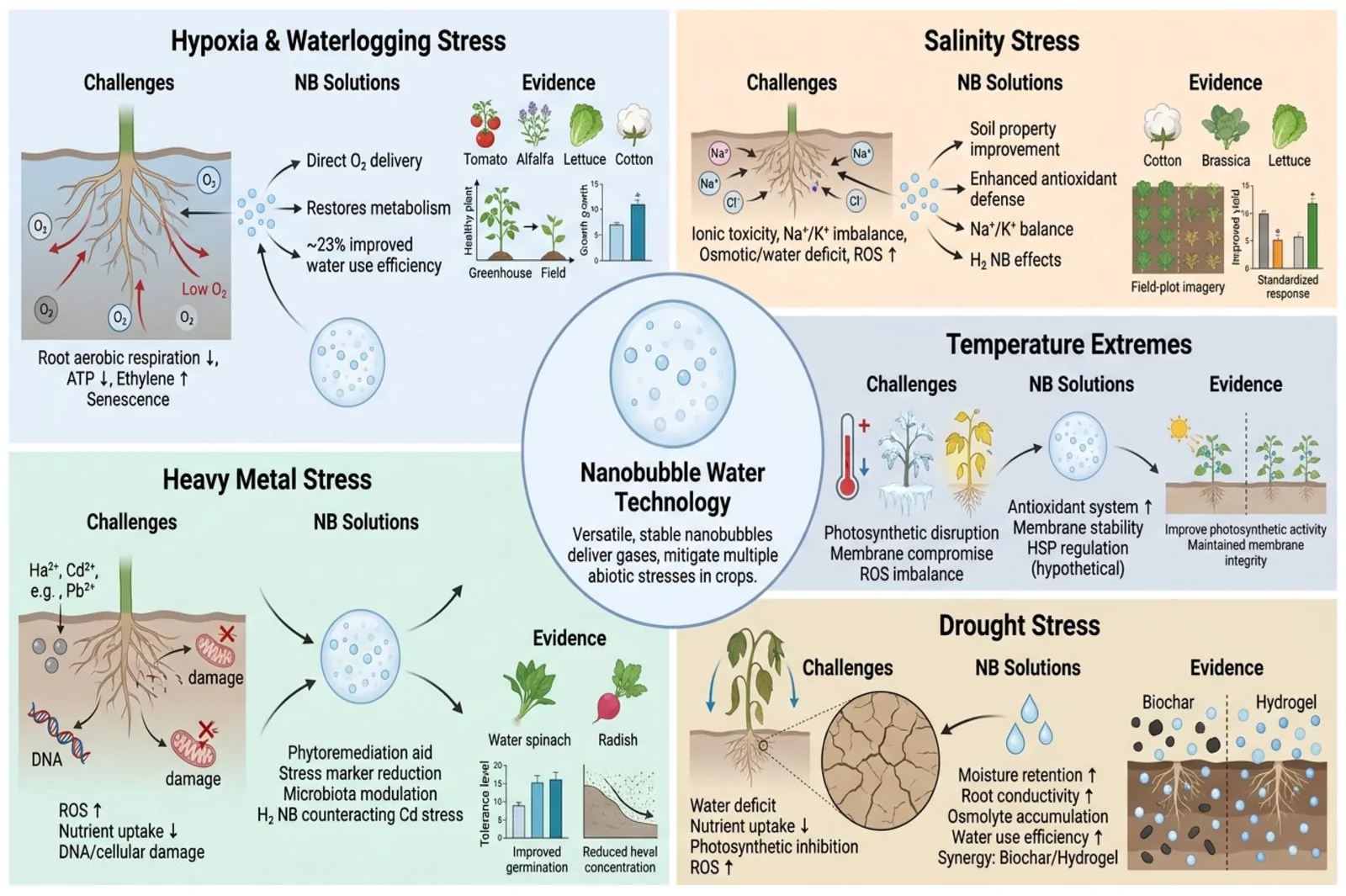
Nanobubble water technology as a mitigation strategy against major abiotic stresses in crops. Upward (↑) and downward (↓) arrows denote increasing and decreasing trends in the respective traits, whereas red cross symbols (✕) indicate inhibition or impairment of the corresponding biological processes.

Figure 5 integrates the evidence discussed in this section into a multi-stress view of NB technology. For hypoxia and waterlogging, the challenges are reduced root aerobic respiration, declining ATP, and rising ethylene and senescence; the NB solutions are direct O_2_ delivery and restoration of metabolism. For salinity, the challenges are ionic toxicity, Na^+^/K^+^ imbalance, osmotic or water deficit, and elevated ROS; the NB solutions are improved soil properties and enhanced antioxidant defense, with evidence in cotton, brassica, and lettuce. For heavy-metal stress (Hg^2+^, Cd^2+^, and Pb^2+^), the challenges are elevated ROS, reduced nutrient uptake, and DNA and cellular damage; the NB solutions are phytoremediation support, reduced stress markers, microbiota modulation, and H_2_-NB counteraction of Cd stress, with evidence in water spinach and radish. For temperature extremes, the challenges are photosynthetic disruption, membrane compromise, and ROS imbalance, which NBs may counter through antioxidant and heat-shock-protein-related mechanisms. For drought, the challenges are water deficit, reduced nutrient uptake, photosynthetic inhibition, and elevated ROS, and the NB solutions are improved moisture retention, membrane stability, root conductivity, and water-use efficiency, with additional synergy reported with biochar and hydrogel.

### 5.5. Seed Priming with Nanobubble Water

Application of NBs during seed imbibition, referred to as NB seed priming, is a very efficient method because of the high oxygen requirement during germination. Ebina et al. [23] already provided evidence that NB water solutions promote plant growth, thus laying a foundation for crop-specific research into priming. Ahmed et al. [59] reported that, in lettuce, carrot, fava bean, and tomato, imbibition in O_2_-NB water raised dissolved oxygen from 7.7 to 31.7 mg L^−1^ and increased germination rates by 6–25%, with N_2_ NBs reported as the most effective, and NB water promoted the growth of barley sprouts, an effect supported by RNA-seq analysis [58]. Mechanistic studies of aquaporin and ABA regulation in NB-primed seeds are still lacking. The physiological basis of NB seed priming effectiveness is that the respiratory need for oxygen during imbibition and radicle emergence is very high, and in compacted, saline, or dry soils the seed depends largely on the O_2_ supply from the environment. Aerobic respiration in the seeds is supported by the O_2_ reservoir that NB priming provides within the water film surrounding the seeds, which will enhance their stress tolerance during the subsequent root development in the crop.

### 5.6. Perspective: Applicability of the NB-Oxygenation Model to C3, C4, and CAM Species

The mechanistic model developed in this review (Figure 4) was derived largely from C3 crops, because most NB studies in Table 1 were conducted on rice, wheat, cotton, tomato, cucumber, and lettuce, all of which fix carbon through C3 photosynthesis. Maize [25] and amaranth (*Amaranthus viridis* L.) [60] are the C4 species represented in Table 1, and, to our knowledge, no NB experiment has yet been reported for an obligate or facultative CAM species.

In C3 species, CO_2_ is fixed directly by Rubisco in mesophyll cells, and net carbon gain is more sensitive than in C4 species to photorespiration and to stomatal closure under heat and water deficit [64,65]. Because stomatal closure under salinity and drought restricts the photosynthate supply to roots (Section 2.2), root respiration in C3 crops is expected to be particularly vulnerable to the coupling between shoot carbon status and root oxygen demand, which is the situation in which an exogenous oxygen source delivered by NBs should be most valuable. Wetland C3 species such as rice can partly buffer the root zone through constitutive aerenchyma and radial oxygen loss barriers [15,66], so the benefit of NBs in rice is expected to be greatest under aerobic or water-saving cultivation (Section 5.1), whereas non-wetland C3 crops without constitutive aerenchyma, such as wheat, cotton, tomato, and cucumber, are likely to depend more strongly on external oxygen supply. C4 species, such as maize, sorghum, and sugarcane, operate a CO_2_-concentrating mechanism between mesophyll and bundle-sheath cells that suppresses photorespiration and increases water-use efficiency, but at an additional energy cost [64,65,67]. Together with the ATP-dependent ion exclusion required under salinity (Section 2.1), this higher energy demand suggests that C4 crops also depend on efficient oxidative phosphorylation in roots, so that NB-derived oxygen would be expected to be beneficial when root-zone oxygen becomes limiting. Maize is sensitive to waterlogging [32] and responded to micro-/nanobubble oxygenation through changes in rhizosphere phosphorus availability [25], but the aquaporin, ABA, and antioxidant responses hypothesized in Section 4 have not been tested in any C4 species. CAM plants fix CO_2_ at night through phosphoenolpyruvate carboxylase, store the resulting malate in the vacuole, and decarboxylate it during the day behind closed stomata, which gives very high water-use efficiency [68,69,70]. In facultative CAM species, the pathway can be induced or enhanced by salinity and water limitation [68,71], which makes these species relevant to the salt- and drought-affected soils targeted by NB irrigation. Because nocturnal carboxylation depends on carbohydrate breakdown and ATP supply at night, nocturnal root and shoot respiration is predicted to be an important oxygen sink, and NB oxygenation may therefore be most useful for CAM crops grown under saline irrigation or in heavy, poorly drained soils. Overall, the available evidence supports the NB-oxygenation model for C3 crops, provides limited support for C4 crops, and provides none for CAM crops. Priority experiments are side-by-side comparisons of C3, C4, and CAM species under identical dissolved oxygen levels, salinity, and soil conditions, including conventionally aerated controls at equivalent dissolved oxygen (Section 8.1), with measurements of root respiration, ATP pools, and the HRG expression described in Section 7.

## 6. Integration of Nanobubble Technology with Nano-Enabled Agriculture

### 6.1. Conceptual Framework: NB Water as a Nano-Agricultural Platform

Along with the evolution of NB water technology, there is a wider shift towards nano-enabled technology in agriculture, such as nanoparticles (NPs), nanofertilizers, nanosensors, nano-biotechnology tools, nanocapsules, and nanocarriers [72,73]. Several complementary properties of NB technology and these approaches are especially relevant: NB water as a carrier medium to root systems; NB oxygen as an energy enabler for NP uptake mechanisms; both NB and engineered NPs have an operating scale of <100 nm, which allows them both to penetrate soil pores, cross root cell walls, and interact with intracellular systems. Nanotechnology has emerged as a transformative field in agriculture, offering innovative solutions to enhance plant growth and resilience against abiotic stresses; by optimizing nutrient delivery, strengthening plant defense mechanisms, and enabling precise monitoring of environmental conditions, it enhances soil quality and regulates physiological responses under environmental stressors [74]. Nano-enabled approaches have emerged as promising tools to control nutrient deficiency, increase crop yields, transform biological systems, and manage plant stresses imposed by the environment [72]. The integration of NB water into this nano-agricultural landscape creates a multi-dimensional intervention platform addressing oxygen delivery, nutrient bioavailability, stress signaling, and rhizosphere ecology simultaneously.

### 6.2. NB Water and Nanoparticle Co-Delivery Systems

The synergistic co-delivery of NB water with engineered NPs represents one of the most promising nano-agricultural frontiers. NB water reduces the surface tension and viscosity of irrigation solutions, enhancing the infiltration rate and uniform distribution of NP suspensions through soil pore networks. The negatively charged NB surfaces may interact electrostatically with positively charged NP surfaces (e.g., ZnO NPs, TiO_2_ NPs), facilitating NP dispersion and preventing aggregation, a major limitation of NP bioavailability in soil systems [72]. Experimental evidence for NB-assisted NP delivery is currently limited to wheat, in which nanobubble watering alleviated the growth and nutrient-status penalties caused by ZnO-NPs [26]. Whether NBs improve the delivery of beneficial NPs in stressed crops, and whether NB–NP combinations produce responses that cannot be predicted from the individual treatments, remain to be tested. Silicon NPs (SiNPs) are conceptually well suited for NB co-delivery: silicon enhances cell wall rigidity and reduces Na^+^ uptake under salinity [72,73], and NB-derived O_2_ could sustain the energy-dependent transport processes involved, although NB–SiNP co-delivery has not yet been tested experimentally. Cerium oxide (CeO_2_) NPs with demonstrated ROS-scavenging activity represent another candidate for NB co-delivery; their catalytic SOD/CAT mimicry could complement the antioxidant priming effect of NB-derived •OH at low concentrations [73].

### 6.3. Nanofertilizers and NB-Mediated Nutrient Delivery

Conventional fertilizer application efficiency is severely compromised under salinity and drought stress because ion competition (Na^+^ displacing K^+^, Ca^2+^, Mg^2+^), soil pH extremes, and root dysfunction all limit nutrient uptake. Nanofertilizers, encapsulated or surface-modified nutrient delivery vehicles in the 1–100 nm size range, address these limitations through slow-release, targeted delivery to the root zone [72]. NB water provides a natural synergistic delivery medium for nanofertilizers: the DO-enriched, low-surface-tension NB solution simultaneously improves the soil physicochemical environment for nutrient availability (EC reduction, pH buffering, aggregate formation), restores root energy status for active nutrient uptake, and provides a liquid matrix that maintains nanofertilizer suspension and distribution [74]. Air injection into paddy soil has been reported to increase nitrogen uptake by 8.3% in rice through improved nitrogen cycling [42], and NB oxygenation enhanced phosphorus availability through alkaline phosphatase stimulation in maize [25], suggesting that soil oxygenation can improve major nutrient cycles independently of co-applied nanofertilizers.

### 6.4. NB Water for Nano-Seed Priming

The convergence of NB seed priming with nano-enabled priming approaches, including hydropriming with NP solutions (ZnO, TiO_2_, CeO_2_) and nanoencapsulated plant growth regulator delivery, represents an emerging precision crop improvement strategy. During seed imbibition, the hypersaturated DO in NB priming solutions meets the high O_2_ demand of germinating embryos, while simultaneously delivering dissolved NPs to the seed coat and embryo surface [59]. Combined oxygen–nanoparticle seed priming has not yet been tested experimentally; NB seed priming alone has been reported to improve germination and early growth in several crop species [23,58,59], whereas nano-priming under salinity stress is supported by the broader nano-enabled agriculture literature [72,73]. The low-input, pre-planting nature of seed priming makes it particularly accessible as an entry point for smallholder farmers in resource-limited settings where continuous field-level NB irrigation may not be economically feasible.

### 6.5. Nano-Biosensors and Smart NB Irrigation Systems

The integration of NB oxygen delivery with real-time nano-biosensor monitoring of root-zone conditions represents the frontier of precision nano-enabled agriculture. Electrochemical DO sensors based on nano-electrode arrays or graphene-modified sensing platforms enable continuous, in situ monitoring of rhizospheric DO concentrations at sub-millimeter spatial resolution, providing the feedback data needed to optimize NB generation rates and irrigation timing to maintain target DO thresholds in the root zone [74]. Ion-selective nanosensors for Na^+^, K^+^, and pH monitoring could further enable real-time tracking of salt-induced ionic stress in the root zone, allowing NB oxygen dosing to be coupled dynamically to ionic stress severity, a precision irrigation concept where the NB oxygen supply is modulated as a function of real-time salinity-hypoxia co-stress status. Dual mechanisms of nanobubble technology for plant growth enhancement and soil remediation, including heavy metals and organic pollutants, have been comprehensively examined, and NB stability and transport behaviors in soil matrices are increasingly well-characterized [75], providing a mechanistic foundation for sensor-guided NB precision delivery.

### 6.6. Environmental Safety and Sustainability Considerations

The environmental profile of NB water technology is generally favorable relative to chemical alternatives (e.g., calcium peroxide as oxygen source), though important considerations warrant evaluation. The controlled •OH generation from NB collapse, while beneficially utilized for ROS priming and antimicrobial effects, has theoretical potential for off-target DNA oxidative damage in sensitive microorganisms at high NB concentrations [36,37]. Long-term soil microbiome stability under repeated NB irrigation requires monitoring: although short-term microbiome enrichment with PGPR is well documented [45], the persistence and ecological stability of NB-driven microbiome shifts across seasons require field-scale investigation. The energy cost of NB generation remains a significant scalability challenge; hydrodynamic cavitation systems achieve the best energy efficiency but still require pump operation at pressures of 200–600 kPa above ambient [20]. Integration of NB generators with renewable energy sources (solar-powered drip irrigation systems) represents a viable pathway to climate-neutral, cost-effective NB irrigation deployment, particularly in arid regions where solar irradiance is high, and salinity stress is severe.

## 7. Multi-Omics Approaches to Decode NB-Mediated Abiotic Stress Tolerance

### 7.1. The Multi-Omics Rationale for NB Research

**Figure 6:**
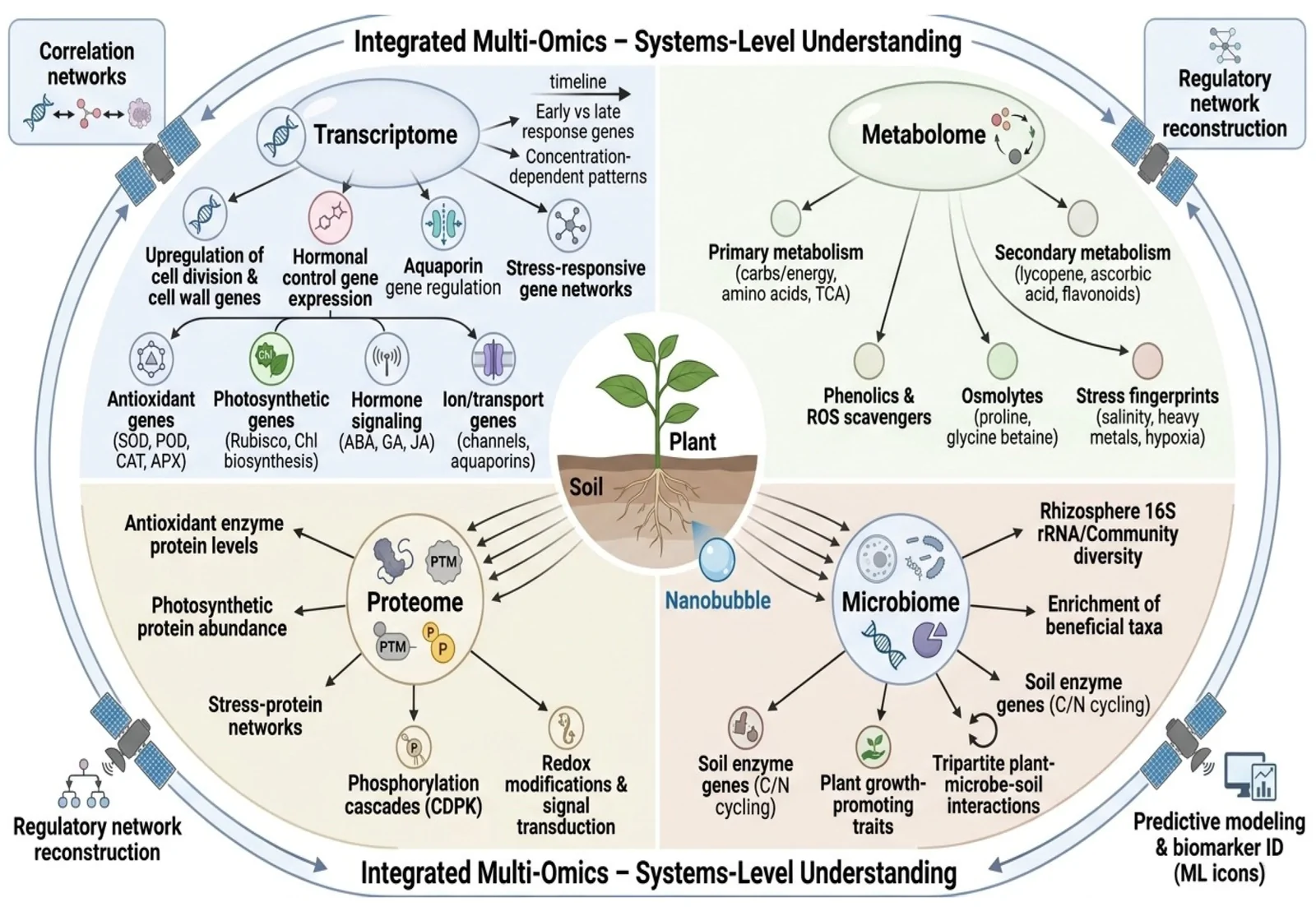
Integrated multi-omics framework for decoding crop responses to nanobubble irrigation. The curved arrows along the outer circular boundary illustrate the integration of transcriptomic, metabolomic, proteomic, and microbiome datasets for systems-level analysis, network reconstruction, and predictive modeling. These arrows show conceptual associations and analytical workflows rather than confirmed causal relationships.

Single-layer analysis, measuring growth parameters, enzyme activities, or expression of a few candidate genes, cannot capture the systemic nature of NB-mediated molecular reprogramming in stressed plants. The molecular consequences of NB irrigation span gene expression networks (transcriptome), protein abundance and modification landscapes (proteome), metabolic flux rerouting (metabolome), ionic rebalancing (ionome), epigenetic reprogramming (epigenome), and whole-plant structural responses (phenome), necessitating a multi-omics integration approach for mechanistic understanding [76,77]. The panomics pipeline, including genomics, transcriptomics, proteomics, metabolomics, epigenomics, proteogenomics, interactomics, ionomics, and phenomics, has become increasingly powerful for characterizing plant traits for abiotic stress response and tolerance mechanisms [78]. Applied to NB water research, this pipeline enables the construction of a comprehensive, systems-level model mapping how NB-delivered O_2_ at the rhizosphere propagates through molecular signaling networks to produce the observed phenotypic improvements in growth, yield, and stress tolerance. The proposed multi-omics framework is shown in Figure 6.

### 7.2. Transcriptomics: Decoding HRG and ERF-VII Network Responses

RNA-sequencing (RNA-seq) applied to NB-treated crop roots under salinity and drought conditions provides the most direct molecular evidence for NB-mediated hypoxia rescue at the transcriptional level. The expected transcriptomic signature of successful NB treatment includes: (i) downregulation of HRGs (ADH1, PDC1, SUS1/4, LBD41, HRE1/2) as ERF-VII proteins are degraded by restored PCO activity; (ii) upregulation of aquaporin gene families (PIP1, PIP2, TIP1 subfamilies) reflecting restored membrane water and gas transport; (iii) upregulation of ion transporter genes (HKT1, SOS1, NHX1) reflecting restored ATP-driven ion homeostasis; and (iv) modulation of ROS-scavenging enzyme gene networks [31,33]. Transcriptome comparisons of lowland and upland rice under root-zone hypoxia [31] provide a reference set of hypoxia-responsive genes that can serve as candidate biomarkers for NB-mediated hypoxia alleviation. In *Arabidopsis*, salt stress transcriptomic studies have defined key transporters, osmoprotectant biosynthesis enzymes, and antioxidant systems across omics levels, identifying principal regulators including transcription factors and protein kinases in the salt stress response network [77]. Integration of multi-omics approaches combining transcriptomics with proteomics and metabolomics has provided evidence about the hierarchical structure of signaling pathways, from stress recognition to downstream events [77]. Advances in high-throughput sequencing have propelled transcriptomics as a pivotal post-genomic discipline, offering unprecedented opportunities to dissect molecular mechanisms underlying stress responses to thermal, saline, water deficit/excess, and heavy metal stresses. Single-cell RNA-seq approaches, now increasingly accessible for crop plants, could further dissect cell-type-specific NB responses in root cortex, endodermis, and meristematic cells, regions with distinct functional roles in oxygen sensing, ion exclusion, and root growth.

### 7.3. Proteomics: Mapping Antioxidant and Fermentation Enzyme Dynamics

Mass spectrometry-based quantitative proteomics provides the functional counterpart to transcriptomics, capturing post-transcriptional modifications, protein stability changes, and enzyme activity regulation that transcript-level data cannot detect. In the context of NB irrigation under abiotic stress, key proteomic targets include: fermentation enzymes (ADH, LDH, PDC) expected to decrease under NB-restored aerobic conditions; antioxidant enzymes (SOD, CAT, APX, GR) expected to show dynamic priming-then-normalization trajectories; ion transporter proteins (SOS1, HKT1, V-H^+^-ATPase subunits) expected to increase under ATP-restored conditions; and aquaporin proteins (PIP, TIP families) expected to increase in abundance and phosphorylation-dependent open-channel states. Proteome reprogramming under stress has been documented, for example, in potato cells exposed to water deficit, where extensive proteome changes were identified that provided insights into metabolic flexibility and stress adaptation pathways [79]. For NB research specifically, iTRAQ- or SILAC-based quantitative proteomics comparing NB-irrigated vs. non-irrigated stressed roots would directly quantify the protein-level magnitude of fermentation enzyme suppression and antioxidant enzyme activation, critical metrics for assessing NB treatment efficacy at the biochemical level. Temporal proteomics (sampling at 1 h, 6 h, 24 h, and 72 h post-NB irrigation) would further reveal the kinetic progression of NB-mediated molecular recovery from hypoxic stress, from initial ATP restoration through ERF-VII suppression to full cellular normalization.

### 7.4. Metabolomics: Tracing Oxygen-Metabolite Flux

Metabolomics, mass spectrometry-based global profiling of low-molecular-weight metabolites, provides the most direct readout of the metabolic consequences of NB-mediated oxygen restoration. Key metabolic signatures of NB-mediated hypoxia rescue include: (i) reduction in ethanol, acetaldehyde, and lactate, fermentation byproducts that serve as quantitative biomarkers of anaerobic catabolism [18]; (ii) increase in TCA cycle intermediates (citrate, malate, succinate) reflecting restored aerobic metabolism; (iii) modulation of osmolyte pools (proline, glycine betaine, GABA) as osmotic adjustment is maintained or enhanced under improved energetic conditions; and (iv) changes in ABA and ethylene metabolite profiles reflecting hormonal rebalancing under restored oxygen conditions. The integration of transcriptomics, proteomics, and metabolomics data has provided a systematic view of the reprogramming of metabolism in plants under stress, defining a group of principal regulators at each omics level [77]. To our knowledge, NB-specific metabolomic datasets for crops under salt–hypoxia stress are not yet available, so the metabolic signatures listed above remain predictions to be tested. Non-targeted metabolomics combined with metabolic flux analysis (using ^13^C-labeled glucose tracers) would provide particularly powerful evidence for NB-mediated shifts between fermentative and oxidative metabolic pathways, directly quantifying the energetic transition from anaerobic to aerobic catabolism at the metabolite level in NB-treated roots.

### 7.5. Epigenomics: Heritable Stress Memory of NB Priming

DNA methylation, histone modification, and small RNA-mediated epigenetic regulation play increasingly recognized roles in stress-responsive gene expression and the “stress memory” phenomenon in plants, where prior stress exposure primes more rapid and robust responses to subsequent stress events [76]. In the context of NB seed priming, which has been reported to improve germination and early growth in several crops [58,59], epigenomics provides a mechanistic framework for understanding how a transient NB oxygen exposure during germination could produce lasting changes in stress gene expression programs throughout the crop’s life cycle. Bisulfite sequencing (WGBS) and ChIP-seq for active histone marks (H3K4me3, H3K27ac) on HRG promoter regions in NB-primed seedlings would directly test whether NB priming leaves an epigenetic imprint—reduced DNA methylation or enhanced activating histone marks at ERF-VII/HRG promoters—that maintains their transcriptional suppression under subsequent stress. Conversely, enhanced methylation at fermentative pathway gene promoters (ADH1, PDC1) following NB priming would represent a form of metabolic epigenetic pre-conditioning that reduces the fermentation response to subsequent hypoxic episodes.

Figure 6 depicts the integrated multi-omics framework proposed for systems-level decoding of crop responses to NB irrigation. The framework is centered on the NB–plant–soil continuum and links four omics layers, each with its NB-responsive features. The transcriptome layer includes upregulation of cell-division and cell-wall genes, hormone-control genes, aquaporin regulation, and stress-responsive gene networks, together with antioxidant (SOD, POD, CAT, and APX), photosynthetic (Rubisco and chlorophyll biosynthesis), hormone-signaling (ABA, GA, and JA), and ion and transport modules, which are resolved across early versus late and concentration-dependent response patterns. The proteome layer covers antioxidant and photosynthetic protein abundance, stress-protein networks, phosphorylation cascades (CDPK), and redox-based post-translational modifications and signal transduction. The metabolome layer distinguishes primary metabolism (carbohydrates and energy, amino acids, and the TCA cycle) from secondary metabolism (lycopene, ascorbic acid, and flavonoids), phenolics and ROS scavengers, osmolytes (proline and glycine betaine), and stress fingerprints (salinity, heavy metals, and hypoxia). The microbiome layer covers rhizosphere 16S rRNA community diversity, enrichment of beneficial taxa, soil enzyme and C–N-cycling genes, plant-growth-promoting traits, and tripartite plant–microbe–soil interactions. Systems-biology integration (correlation networks, regulatory network reconstruction, predictive modeling, and biomarker identification with machine learning) unifies these layers into a mechanistic understanding of NB action, as developed in Section 7.2, Section 7.3, Section 7.4, Section 7.5, Section 7.6, Section 7.7 and Section 7.8.

### 7.6. Ionomics: NB-Mediated Ion Profile Restoration

Ionomics, the comprehensive profiling of elemental concentrations in plant tissues by ICP-MS or ICP-OES, provides a direct quantitative measure of NB treatment’s impact on ionic homeostasis under salinity stress. The expected ionomic signature of successful NB irrigation under salinity includes: (i) reduced Na^+^ and Cl^−^ concentrations in root and shoot tissues, reflecting restored SOS-pathway-driven Na^+^ extrusion; (ii) increased K^+^/Na^+^ ratio in root cells, reflecting enhanced K^+^ retention through HKTs; (iii) normalized Ca^2+^ and Mg^2+^ concentrations reflecting restored divalent cation uptake; and (iv) reduced Fe^2+^ accumulation, as aerobic conditions under NB irrigation convert soluble Fe^2+^ to insoluble Fe^3+^, reducing free iron availability for Fenton-chemistry ROS generation [35]. Elemental profiling evidence for NB treatment is still preliminary; for example, NB treatment altered cadmium uptake and accumulation in rice seedlings [49] and was associated with upregulation of nutrient-uptake genes (OsBT, PiT-1, and SKOR) [24]. More comprehensive ionomic profiling across root, shoot, and grain tissues in NB-treated vs. control crops would provide a complete picture of NB’s systemic effects on plant mineral nutrition under stress, information critical for assessing the crop quality and nutritional implications of NB irrigation at commercial scale.

### 7.7. Phenomics: High-Throughput Plant Phenotyping for NB Evaluation

High-throughput plant phenotyping, using imaging, spectroscopy, and sensor platforms to measure plant structural, functional, and physiological traits at scale, provides the bridge between molecular-level NB mechanisms and agronomic outcomes [76]. For NB irrigation research, key phenomic parameters include: root architecture analysis (root length, surface area, branching density via rhizotron imaging or CT scanning); chlorophyll fluorescence imaging (Fv/Fm, ΦPSII) to assess photosynthetic efficiency recovery; hyperspectral canopy reflectance for detecting water content and chlorophyll changes; and thermal imaging for stomatal conductance mapping as an indicator of improved plant water status under NB irrigation. Harnessing multi-omics and genome-editing technologies for climate-resilient agriculture, bridging AI-driven insights with sustainable crop improvement, is an emerging frontier [76] where NB-mediated molecular changes could provide critical intervention points identified through integrated phenomic-genomic analysis. The application of machine learning to multi-omics data from NB irrigation experiments, particularly neural network models trained on transcriptomic + metabolomic + phenomic data, could enable predictive modeling of NB dose–response relationships across different crop genotypes, soil types, and stress severities, ultimately enabling AI-guided precision NB irrigation scheduling.

### 7.8. Integrative Multi-Omics: Systems-Level Model of NB Action

The construction of a comprehensive, systems-level model of NB water action in stressed crops requires the integration of data from all omics layers, from transcriptome through proteome through metabolome through ionome and epigenome, into network-based models that reveal the hierarchical organization of NB-responsive molecular pathways. In rice, the combined use of RNA-seq, GWAS/QTL mapping, and metabolomics has enabled the discovery of drought and salt-tolerance genes, improved marker-assisted selection and facilitating the development of elite stress-resilient lines [78]. Applied to NB research, a similar multi-omics integration strategy would: (i) identify core NB-responsive gene modules through co-expression network analysis; (ii) connect transcriptomic nodes to proteomic and metabolomic endpoints through stoichiometric metabolic flux models; (iii) map the temporal sequence of NB-mediated molecular events (from first O_2_ delivery to full cellular normalization) using time-series multi-omics; and (iv) identify NB-specific molecular markers (transcripts, proteins, metabolites) that could serve as precision biomarkers for monitoring NB treatment efficacy in field conditions.

## 8. Knowledge Gaps, Challenges, and Future Directions

### 8.1. Mechanistic Gaps

Despite substantial progress, several critical mechanistic questions remain unresolved: Direct molecular evidence for NB-driven ERF-VII suppression in crops: The mechanistic link between NB oxygen delivery and ERF-VII degradation has been inferred from oxygen concentration effects on PCO activity and from animal HIF-1α studies [43], but has not been directly demonstrated in crop plants using either ERF-VII protein quantification or ERF-VII target gene (HRG) expression analysis under controlled NB treatment experiments. NB-specific vs. bulk-DO effects: It is unclear whether the biological effects of NB water are attributable solely to elevated dissolved oxygen content or whether NB-specific properties (surface charge, •OH generation, surface tension modification, physical interaction with root surfaces) contribute independently to the observed stress mitigation. Controlled comparison of NB water with conventionally aerated water at equivalent DO concentrations would directly test this question. Long-term effects on aerenchyma and root architecture: Whether NB oxygen delivery, by suppressing hypoxia-driven ERF-VII signaling, also reduces protective aerenchyma formation and thereby increases vulnerability to subsequent hypoxic episodes, remains an important unanswered question with significant practical implications. Epigenetic memory of NB priming: The duration, stability, and transmissibility across plant generations of epigenetic changes induced by NB seed priming have not been investigated, a critical gap for understanding whether NB priming effects persist throughout the crop growth cycle and whether they have implications for future crop improvement programs. Photosynthetic-type specificity: It is unknown whether the NB responses described for C3 crops are conserved in C4 and CAM species, which differ in energy demand, water-use strategy, and root–shoot carbon coupling (Section 5.6).

### 8.2. Technological Challenges

Energy cost at the field scale: The energy needs of NB generation are still a major barrier to scaling up the technology. The most energy-efficient approach is hydrodynamic cavitation, but it still requires a considerable amount of pump energy at the field scale. The challenges of integrating with renewable energy sources and optimizing NB generation efficiency are priority engineering challenges [20]. DO stability during irrigation distances: NB water’s DO decreases with distance in irrigation pipelines and soil profiles, especially at high temperatures. The placement of NB generators, pipeline insulation, and injection frequency must be optimized for large fields to maintain target DO concentrations at root delivery points. Limited NB characterization comparability and reproducibility: The lack of standardization in the characterization of NB (size distribution, concentration, DO content) across research groups diminishes the comparability and reproducibility of the agricultural studies of NB. Development and adoption of standardized NB analytical protocols is a prerequisite for evidence-based field deployment guidelines. Crop and soil specificity: NB effects are reported to be highly dependent on crop species, soil type, stress level, optimum DO concentration, irrigation frequency, and the timing of NB application, which are expected to differ among rice paddies, upland maize, drip-irrigated vegetable crops, and cotton in saline-alkali soils [27,40].

## 9. Conclusions

This review has constructed a comprehensive, mechanistic account of how nanobubble water technology, through precision delivery of supersaturated dissolved oxygen to the rhizosphere, addresses the convergent downstream consequence of salinity and drought stress that has been critically underappreciated in the abiotic stress literature: root-zone hypoxia. By restoring the cellular oxygen supply that powers aerobic respiration, NB-delivered O_2_ simultaneously rescues ATP homeostasis, suppresses the ERF-VII/HRG fermentation cascade, reactivates energy-dependent ion homeostasis machinery, upregulates aquaporins and hydraulic conductivity, modulates the ABA-ethylene hormonal cross-talk that governs water stress responses, and restores the rhizosphere microbiome, addressing hypoxic injury at every level from molecular signaling to ecosystem function. The combination of NB water technology with nano-enabled agricultural approaches (NP co-delivery, nano-formulated fertilizers, and nano-biosensor-controlled irrigation) provides a synergistic precision-agriculture platform that addresses abiotic stress comprehensively rather than as isolated stress components. The new multi-omics methodology, spanning transcriptomics, proteomics, metabolomics, epigenomics, ionomics, and phenomics, will provide the tools to understand NB-mediated molecular responses at systems-level resolution and to identify specific biomarkers, regulatory genes, and metabolic networks underlying the stress-protective effects of NB on crop plants. The novelty of this review lies in the first explicit mechanistic link that bridges three research fronts, namely hypoxia rescue by NB, integration of agriculture with nanotechnology, and multi-omics decoding, within a unified concept of climate-smart crop stress management. A perspective on photosynthetic types further suggests that the oxygen-supply benefit of NBs should extend beyond C3 crops, although the magnitude of the response in C4 and CAM species remains to be tested. As global soil salinization intensifies and drought frequency escalates under climate change, NB water technology offers a chemical-free, energetically scalable, and mechanistically grounded approach to sustaining crop productivity in degraded agricultural environments, provided that the identified research gaps in molecular mechanism, field-scale validation, and socioeconomic feasibility are systematically addressed.

## Figures and Tables

**Table 1:** Synthesis of empirical nanobubble (NB)/micro-nanobubble (MNB) studies relevant to abiotic stress–induced hypoxia in crops: gas type, application mode, mechanistic and multi-omics effects, and agronomic outcomes.

Crop/Plant Species	Bubble Type and Gas	NB Property/DO Level	Abiotic Stress Condition	Application Mode	Key Mechanism and Molecular/Multi-Omics Effects	Quantitative Outcome	Study
Rice (*Oryza sativa* L.)	NB water (air/O_2_)	High colloidal stability	None (nutrient-use optimization)	NB irrigation (lab + field)	Upregulated nutrient-uptake genes (OsBT, PiT-1, SKOR); stimulated GA biosynthesis; enhanced root elongation	Equal yield with ~25% less fertilizer; ↑ plant height and root length	[24]
Rice (*Oryza sativa* L., salt-tolerant cultivar JX99)	Micro-/nanobubble water (O_2_)	—	0.6% NaCl salt stress	Hydroponic/NB oxygenation	Reduced membrane lipid peroxidation (↓ MDA); modulated proline; salt-tolerant JX99 most responsive	JX99 stem/root length and fresh weight ↑ 28–68% under salt	[48]
Rice (*Oryza sativa* L.)	Air/O_2_/N_2_ nanobubbles	—	Cadmium (Cd) toxicity	Hydroponic culture	Different NB gases mitigated Cd toxicity and root uptake; modulated antioxidant response	Reduced Cd accumulation and phytotoxicity in seedlings	[49]
Rice (*Oryza sativa* L.), cv. Huruan1212	H_2_ NB water	—	Field grain quality and Cd accumulation; no imposed salt–hypoxia treatment	Field irrigation versus ditch water	Grain trait changes and altered expression of Cd transporter genes; qPCR association does not establish each transporter as causal	White-rice Cd reached 52% of control (about 48% lower); grain dimensions and 1000-grain weight increased	[51]
Rice (*Oryza sativa* L.)	O_2_ NBs	DO elevated after NB generation (value not reported)	Continuously flooded paddy soil; reducing conditions and As dissolution	Pot irrigation with NB water versus control water	Maintained a more oxidizing soil environment; investigated methane emissions and dissolved arsenic rather than salt tolerance	Seasonal cumulative methane emission −21%; rice growth did not differ from control	[50]
Maize (*Zea mays* L.)	Micro-/nanobubble oxygenation (O_2_)	—	Field conditions (no imposed stress)	Oxygenated irrigation versus conventional irrigation	Altered bacterial and fungal community networks; ↑ rhizosphere alkaline phosphatase activity	Rhizosphere available P ↑ 21.3%; alkaline phosphatase activity ↑ 15.4%	[25]
Wheat (*Triticum aestivum* L.)	Nanobubble water + ZnO nanoparticles	Mean size about 74 nm (manufacturer specification)	ZnO-NP phytotoxicity (500 mg L^−1^)	NB irrigation of seedlings	Improved foliar N and P status, photosynthesis, and antioxidant capacity under ZnO-NP stress	Alleviated ZnO-NP-induced growth penalty (higher shoot fresh and dry weight)	[26]
Alfalfa (*Medicago sativa* L.)	Micro-nanobubble oxygenation (O_2_)	—	None (irrigation amount × oxygen level)	Subsurface drip irrigation with oxygenation	↑ Soil enzyme activities and microbial abundance; rhizosphere mechanisms resolved by structural equation modeling	Higher yield; catalase ↑ 15–28%, urease ↑ 18–32%	[14]
Cotton (*Gossypium hirsutum* L.)	Micro-/nanobubbles (O_2_) + microbial agents	—	Saline-alkali soil	Field micro-plot NB oxygenation irrigation	↓ Soil salinity 26.08%; activated urease, sucrase, dehydrogenase and alkaline phosphatase; ↑ bacterial Chao1; promoted root growth, LAI and dry matter	Seed cotton yield 6278.65 kg·hm^−2^ (+44.53% vs. CK)	[40]
Cotton (*Gossypium hirsutum* L.)	Oxygen-enriched MNBs	Irrigation DO 30 mg L^−1^; source water 8–9 mg L^−1^	Soil salt 0, 3, 6, and 9 g kg^−1^	Field micro-plots; drip delivery to root zone	Salt leaching, altered rhizosphere microbiota, higher SOD/POD, and lower MDA	Seed cotton yield +33.78%, +35.93%, +47.11%, and +52.31%, respectively, versus corresponding conventional-irrigation treatments	[27]
Lettuce (*Lactuca sativa* L.)	Micro-/nanobubble hydrogen water (H_2_)	—	NaCl salt stress	Greenhouse pots; subsurface drip irrigation	Higher antioxidant activity and osmolytes; improved ion homeostasis and photosynthesis; altered hormone levels and soil bacterial community	Fresh weight +148.8% and leaf number +146.4% versus NaCl-only irrigation	[52]
Lettuce (*Lactuca sativa* L.)	Micro-/nanobubbles (O_2_/O_3_)	↑ DO	None (hydroponic)	Nutrient-solution aeration	↑ Net photosynthetic rate, water conductivity and intercellular CO_2_	Improved yield and physiological parameters	[53]
Butterhead lettuce (*Lactuca sativa* L.)	Air MNBs	Grow-bed DO 9.31 ± 1.57 versus 6.32 ± 0.32 mg L^−1^ with diffuser	Aquaponic oxygen-supply limitation	Continuous aeration in floating-raft grow bed	16S rRNA metabarcoding, water chemistry, and predicted functions: altered microbiota, higher nitrification and remineralization	Total lettuce yield +35 ± 8%; root biomass per plant +20 ± 5%, versus conventional diffuser aeration	[46]
Tomato (*Solanum lycopersicum* L.) and cucumber (*Cucumis sativus* L.)	Micro-/nanobubble water oxygenation (O_2_)	DO 15 and 25 mg L^−1^	Greenhouse root-zone O_2_ deficit	Subsurface drip irrigation	↑ Rhizosphere urease and phosphatase; ↑ microbial species; DO 15 at fruiting stage optimal	↑ Yield, IWUE, vitamin C and tomato lycopene	[54]
Tomato (*Solanum lycopersicum* L.)	Nanobubble water oxygenation (O_2_)	NBs were 136.2 ± 12.1 nm and 6.2 ± 108 bubbles mL^−1^	Greenhouse cultivation with drip laterals	Subsurface drip irrigation	↑ tomato yield, WUE, soluble sugar, and vitamin C by NBs, ↓ oxygen concentration in irrigation water. The total C (TC), organic matter, available N, and K ↑ and ↓ with TOC in irrigation water. ↑ NB220 soil OM and AN value, and ↑ NB250 TC and AK values.	Increased abundance of taxa participating in soil nutrient, improved soil fertility, and crop agronomic performance	[55]
Tomato (*Solanum lycopersicum* L.) and cucumber (*Cucumis sativus* L.)	MNB water oxygenation (O_2_)	—	Greenhouse cultivation	Subsurface drip irrigation	↑ Dry-matter accumulation via enhanced root aerobic respiration	Tomato yield +7.8–26.9% (DM +29.2–67.5%); cucumber yield +2.3–16.3% (DM +80.8–108.9%)	[56]
Tomato (*Solanum lycopersicum* L.)	Nanobubble oxygenation (O_2_/air)	—	Organic farming	NB-aerated irrigation	↑ Plant-available N and P release; ↑ soil enzyme and microbial functional diversity	Biomass +22% (≈547 vs. ≈447 g plant^−1^)	[47]
Tomato (*Solanum lycopersicum* L.)	H_2_ NBs versus O_2_ NBs and groundwater	Not reported	Fruit antioxidant enrichment; no imposed abiotic stress	Drip irrigation	Transcriptomics and metabolomics associated hormone/hydrogenase gene changes with synthesis of fruit antioxidants	Across lycopene, ascorbate, flavonoids, and resveratrol: +16.3–264.8% versus groundwater; +2.2–19.8% versus O_2_ NB water	[57]
Strawberry (*Fragaria* × *ananassa* Duch.)	Micro-/nanobubble water (O_2_)	—	Field (with K fertigation)	MNB + K fertigation/SDI	Modulated soil bacterial community structure and nutrient cycling	Improved yield and fruit quality	[21]
Barley (*Hordeum vulgare* L.)	NB water (N_2_/air)	DO matched to control	Sprouting/early growth	NB soaking	Moderate exogenous ROS promoted metabolism; RNA-seq confirmed gene-level growth promotion	Sprout growth +15–25% vs distilled water	[58]
Lettuce (*Lactuca sativa* L.), carrot (*Daucus carota* L.), fava bean (*Vicia faba* L.), tomato (*Solanum lycopersicum* L.)	Air/O_2_/N_2_/CO_2_ nanobubbles	O_2_ NB raised DO 7.7 → 31.7 mg L^−1^	Seed germination	NB-water imbibition	Exogenous ROS generation; improved nutrient fixation/utilization; N_2_ NBs most effective	Germination 6–25% higher across tests; N_2_ strongest; air and CO_2_ did not significantly promote germination	[59]
Amaranth (*Amaranthus viridis* L.)	CO_2_/air micro-/nanobubbles	—	None (hydroponic)	MNB-treated water	Enhanced photosynthetic substrate supply; improved growth and nutritional quality	Statistically significant growth promotion	[60]
Alligator weed (*Alternanthera philoxeroides* (Mart.) Griseb.)	Air nanobubbles	25% vs. 100% NB (dose-dependent)	Cadmium contamination	Air-NB watering	25% air NB ↑ SOD, CAT, POD, APX and soluble protein; 100% NB had a negative effect (concentration threshold)	25% air NBs increased Cd uptake by 17.39%; growth +25–50% and pigment content +10–20% at 0.1 mM Cd	[61]
Water spinach (*Ipomoea aquatica* Forssk.)	H_2_ MNB water	—	Soil Cd 4 mg kg^−1^	Greenhouse pots; subsurface drip irrigation	Higher metallothioneins, ABA, and SA; improved ROS scavenging and rhizosphere properties; lower plant Cd uptake	Improved yield, photosynthesis, and root traits under Cd stress; lower Cd uptake	[62]
Pakchoi (*Brassica chinensis* L.)	H_2_ NBs	Median 175.1 nm; 3.4 × 10^7^ bubbles mL^−1^; initial H_2_ 1.2 ± 0.08 ppm	NaCl 150 mM, reached gradually	Hydroponics; physiological/omics sampling at day 3; growth at day 7	Transcriptomics and metabolomics: rebalanced JA/ABA/ET pathways, phenylpropanoid metabolism, antioxidant enzymes, and soluble sugars	H_2_-NB versus salt-only: 2350 differentially expressed genes and 166 differential metabolites; growth suppression alleviated	[63]

Abbreviations: NB, nanobubble; MNB/ micro-nano/; DO, dissolved oxygen; IWUE, irrigation water-use efficiency; LAI, leaf area index; DM, dry matter; CK, control; SDI, subsurface drip irrigation; MDA, malondialdehyde; ROS, reactive oxygen species; GA, gibberellic acid; ABA, abscisic acid; SOD, superoxide dismutase; CAT, catalase; APX, ascorbate peroxidase; POD, peroxidase; ZnO NP, zinc-oxide nanoparticles. Upward (↑) and downward (↓) arrows denote increasing and decreasing trends in the respective traits.

## Data Availability

No new data were created or analyzed in this study. Data sharing is not applicable to this article.
